# Epigenetic and mitoepigenetic regulation in cancer and therapeutic perspectives

**DOI:** 10.3389/fphar.2026.1760013

**Published:** 2026-02-27

**Authors:** Selcen Celik-Uzuner, Ihsan Nalkiran, Ugur Uzuner, Hatice Sevim Nalkiran

**Affiliations:** 1 Department of Molecular Biology and Genetics, Faculty of Science, Karadeniz Technical University, Trabzon, Türkiye; 2 Department of Medical Biology, Faculty of Medicine, Recep Tayyip Erdogan University, Rize, Türkiye; 3 Pros Biotechnology Trading Ltd., Trabzon Technology Development Zone Management Inc., University District, Ortahisar, Trabzon, Türkiye

**Keywords:** cancer therapy, DNA methylation, epigenetics, histone modification, mitoepigenetics

## Abstract

Epigenetic modifications on nuclear and mitochondrial DNA constitute key regulatory layers influencing the transcriptional, metabolic, and phenotypic adaptability of cancer cells. The canonical principles of epigenetic control encompass DNA methylation, histone modification, and non-coding RNA–mediated regulation, which collectively contribute to the silencing of tumor suppressor genes, the activation of oncogenes, and chromatin remodeling. Therefore, epigenetic drugs (epi-drugs) are of great interest in the development of new-generation therapeutics and holistic treatment approaches. Accordingly, this work presents a narrative review that integrates current evidence on the molecular mechanisms, therapeutic developments, and translational relevance of epigenetic and mitoepigenetic regulation in cancer. RNA–mediated regulation collectively contributes to the silencing of tumor suppressor genes and to the activation of oncogenes. The field of mitoepigenetics encompasses mitochondrial DNA (mtDNA) methylation, RNA modifications, and post-translational regulation of mitochondrial proteins such as TFAM, DNMT1, and sirtuins, which influence oxidative phosphorylation, redox balance, and apoptotic pathways, thereby affecting tumor initiation, progression, and treatment response. Recent advances in epigenetic drug development include FDA-approved DNMT and HDAC inhibitors and newer agents targeting EZH2, IDH1/2, and DOT1L, which broaden the scope of precision oncology. In addition, modulation of mitochondrial epigenetic mechanisms has been identified as a potential approach for addressing metabolic reprogramming and therapeutic resistance in cancer. The convergence of nuclear and mitochondrial regulatory frameworks reveals the critical need for biomarker-informed, combinatory, and organelle-targeted therapeutic approaches to sustain treatment efficacy. Comprehensive characterization and pharmacological targeting of epigenetic and mitoepigenetic networks provide a structured basis for developing personalized and metabolism-informed interventions in cancer therapy.

## Introduction

Epigenetics is the field of investigating the principles of gene regulation without changes in DNA sequence. It primarily involves the reversible chemical modification of DNA and histone proteins, catalyzed by specific enzymes. The main modification of DNA is the methylation of cytosine bases (5′-methylcytosine, 5meC) in CpG (cytosine-guanine repeats) and non-CpG regions; however, hydroxymethylation (5-hydroxymethylation, 5hmC), formylation (5-formyl-cytosine, 5fC), and carboxylation of cytosines (5-carboxy-cytosine, 5caC) are also reported (Hardwick et al., 2018). DNA methylation, the leading epigenetic modification, plays a critical role in the up- or downregulation of genes, depending on the location of methylation on promoters or gene bodies. Promoter hypermethylation is commonly associated with the downregulation of the genes; however, the methylation occurring on gene bodies is associated with upregulation of gene expression. DNA hydroxymethylation is specifically found in neuron cells, suggesting that 5hmC is crucial for the central nervous system (Breiling and Lyko, 2015). 5hmC, 5fC, and 5caC have been reported to be involved in active gene expression (Wang et al., 2015).

The pattern of DNA methylation is crucial for establishing cell fate in early embryogenesis. Each parental nucleus undergoes epigenetic reprogramming immediately after fertilization, before the fusion of maternal and paternal nuclei. While a rapid decrease in DNA methylation occurs in paternal DNA, the profile of maternal DNA methylation gradually decreases with each round of DNA replication during early embryonic development (Eckersley-Maslin et al., 2018). Further cell differentiation is defined by the unique patterns of DNA methylation in each cell type. Therefore, cells with the same genome form specialized tissues and organs with diverse gene expression patterns and function differently. The epigenetic paradigm suggests that the proper regulation of epigenetics is compulsory for life.

Histone proteins are also the pivotal targets of epigenetic modifications, including methylation, acetylation, phosphorylation, *etc*. Acetylated histone tails are related to open chromatin formation, resulting in active gene expression (Nitsch et al., 2021). However, methylation of histone tails is associated with both gene inactivation and activation, depending on the type and location of the modified residue and the type of histone protein. The phenomenon of “histone code”, representing the perspective of post-translational modifications of histones, defines the activity of genes by chromatin remodeling (Rando, 2012). There is also cross-talk between histone and DNA modifications, which adds further complexity to understanding the principles of gene regulation from the epigenetic perspective. The epigenetic operation also includes the function of non-coding RNAs. This specific group of RNAs can manipulate gene expression in a post-transcriptional manner. MicroRNAs are the leading group to inhibit synthesized mRNAs and, therefore, prevent their translation (Shang et al., 2023).

Methylation may occur on both nuclear and mitochondrial DNA. A specialized field of epigenetics for mitochondrial DNA (mtDNA) is called “mitoepigenetics” (Cao et al., 2021). mtDNA is a small and circular DNA that regulates specific mitochondrial functions such as generating and eliminating reactive oxygen species, energy production, and other metabolic activities (Czarna and Jarmuszkiewicz, 2006). mtDNA is small, but the cellular content of mtDNA is relatively extensive due to the total number of mitochondria, which includes around 2-10 copies of mtDNA. This may lead to significant mtDNA methylation within a cell (Celik Uzuner, 2020). mtDNA methylation is supposed to play a role in programming during gametogenesis (Sirard, 2019). The abnormal changes in mtDNA methylation are associated with pathological conditions, such as cancer and neurodegenerative diseases. For instance, abnormal mtDNA methylation is linked to mitochondrial dysfunction, which is a critical factor in the pathogenesis of Alzheimer’s Disease. In particular, the imbalance of mtDNA methylation and demethylation can damage the mitochondrial electron transport chain and obstruct mitochondrial biogenesis (Liu et al., 2022). Methylation changes can lead to reduced expression of essential mitochondrial genes, impairing processes such as ATP production and increasing oxidative stress (Song et al., 2022).

mtDNA is not naked, since it is formed in the nucleoid with some proteins. However, the nucleoid structure is not as complex as the chromatin structure of nuclear DNA. This structure involves the possible interaction between mitochondrial transcription factor A (TFAM), mitochondrial polymerase γ (POLG), ATPase family AAA-domain-containing protein 3 (ATAD3), mitochondrial AAA protease (LONP1), and mitochondrial single-stranded DNA-binding protein (mtSSB) with the D-loop region of mtDNA (Dong et al., 2020). Mitochondrial transcription factor A regulates the gene expression potential of mtDNA, and epigenetic modifications on TFAM proteins are able to affect mtDNA packaging similarly to histones around nuclear DNA. These modifications include acetylation, phosphorylation, and ubiquitination (Santos et al., 2014; King et al., 2018; Dinardo et al., 2003). Mitochondrial RNA molecules are also modified by epigenetic mechanisms (Bar-Yaacov et al., 2016). Therefore, it can be concluded that mtDNA is metaphorically a small nuclear DNA sharing many biological functions and mechanisms. The epigenetic landscape of mtDNA is thus worth being considered as a cellular target in disease and therapy.

Given the emerging and rapidly evolving nature of mitoepigenetics, this article was intentionally designed as a narrative review rather than a systematic review. Literature was identified through searches of PubMed, Web of Science, and Scopus databases, with particular emphasis on critically examining recent advances, while also incorporating earlier foundational studies that established core epigenetic and mitochondrial concepts. Selection criteria prioritized mechanistic relevance, translational significance, and experimental rigor in *in vitro*, *in vivo*, and early-phase clinical studies. Review articles were included selectively to contextualize key concepts, and no formal exclusion based on study design was applied, consistent with the narrative scope of this review.

## Advancements in conventional, targeted, and epigenetic cancer therapies

### Conventional cancer therapies

Conventional cancer therapies, including surgery, radiation therapy, and chemotherapy, have been the cornerstone of cancer treatment for decades. While effective, they often suffer from limitations such as systemic toxicity, lack of specificity, and potential resistance (Liu et al., 2024a). Recent research has focused on refining these approaches to improve their efficacy and reduce potential side effects. Conventional therapies include surgery, radiation, and chemotherapy.

Surgery remains the gold standard for treating localized cancers. Advances in minimally invasive techniques, such as robotic-assisted surgery, laparoscopic procedures, and fluorescence-guided surgery, have improved surgical precision and reduced complications (Nagaya et al., 2017). However, the risk of incomplete tumor excision and disease recurrence remains a challenge, particularly in cases with micrometastases (Liu et al., 2024b).

Radiation therapy employs high-energy ionizing radiation to induce DNA damage in cancer cells, leading to apoptosis (Liu et al., 2021). Technological innovations, such as proton beam therapy and stereotactic body radiation therapy, have significantly enhanced targeting accuracy while minimizing collateral damage to healthy tissues (Koka et al., 2022). Despite these advances, concerns about long-term complications, including radiation-induced fibrosis and secondary malignancies, persist (Fijardo et al., 2024).

Chemotherapy remains a key component of cancer treatment, particularly for metastatic diseases (Ganesh and Massagué, 2021). Recent efforts have focused on optimizing drug combinations to alleviate resistance and enhance efficacy (Hu et al., 2024). Emerging studies suggest that integrating microRNAs (miRNAs) with chemotherapy can modulate drug resistance in cancers such as breast and colorectal malignancies (Hu et al., 2024). Nonetheless, systemic toxicity, including oxidative damage, myelosuppression, and cardiotoxicity, remains a major drawback (Katanić Stanković et al., 2023).

### Targeted cancer therapies

Targeted therapies offer a more precise approach to cancer treatment by interfering with specific molecular pathways that drive tumor progression. These therapies typically involve monoclonal antibodies (mAbs), small-molecule inhibitors, and immune checkpoint inhibitors (Min and Lee, 2022). Targeted therapies function by inhibiting key signaling pathways responsible for cancer cell proliferation, angiogenesis, and immune evasion. These include tyrosine kinase inhibitors (TKIs), monoclonal antibodies, and immune checkpoint inhibitors (ICIs) (Nouri et al., 2020; Li et al., 2024).

Over the past two decades, cancer therapy has broadly evolved from cytotoxic agents toward target-specific and immune-modulatory approaches that reshape intracellular signaling and cell-death pathways. The principal classes of these therapies include mAbs that selectively block extracellular receptors or ligands; TKIs that interfere with oncogenic kinase signaling; and ICIs that restore antitumor immunity. Targeted therapies have led to significant improvements in cancer treatment. For example, trastuzumab has revolutionized the management of HER2-positive breast cancer by specifically inhibiting HER2 signaling (Swain et al., 2023). Similarly, pembrolizumab has emerged as a standard treatment for metastatic melanoma and lung cancer (Goldberg et al., 2016). In addition, two rapidly emerging modalities, including antibody-drug conjugates (ADCs) and chimeric antigen receptor T cell (CAR-T) therapies, represent hybrid or cell-based strategies that combine precise target recognition with potent effector mechanisms (Puppi et al., 2025). While ADCs directly deliver cytotoxic agents to tumor cells and often trigger mitochondria-mediated intrinsic apoptosis (Wang et al., 2025), CAR-T therapies utilize patient-derived or engineered T cells (Pourzia et al., 2023). The health of mitochondria and the metabolic reprogramming of these T cells are essential for their durability and their capacity to eradicate cancer cells (Rad et al., 2021). Together, these therapeutic categories illustrate the expanding therapeutic landscape that indirectly or directly intersects with mitochondrial signaling (Table 1).

**TABLE 1:** Overview of FDA-approved and emerging targeted cancer therapies.

Category	Drug name	Original approval date	Target	Use	References
1. Monoclonal Antibodies (mAbs)	Amivantamab-vmjw (Rybrevant)^a^	20 Feb 2025 (expanded); 21 May 2021	EGFR, MET	NSCLC (EGFR exon 19 del/L858R, exon 20 insertions)	Shah et al. (2023)
	Rituximab (Rituxan)	26 Nov 1997	CD20	NHL, CLL	Grillo-López et al. (2000)
	Trastuzumab (Herceptin)	25 Sep 1998; 19 Jan 2023 (expanded)	HER2	HER2+ breast cancer; HER2+ colorectal cancer (2023)	Dillman (1999)
	Cetuximab (Erbitux)	12 Feb 2004; 27 Jun 2022 (expanded)	EGFR	Head/neck cancer, KRAS wt colorectal cancer; BRAF V600E CRC (2022)	Bonner et al. (2006), Kopetz et al. (2025)
	Bevacizumab (Avastin)	26 Feb 2004	VEGF	Metastatic colorectal cancer, NSCLC, glioblastoma, RCC	Johnson et al. (2004), Yang et al. (2003), Kabbinavar et al. (2003), Gilbert et al. (2014)
	Zolbetuximab^a^ (Vyloy)	18 Oct 2024	Claudin 18.2	Gastric/GEJ adenocarcinoma (CLDN18.2+, HER2-)	Lordick et al. (2024)
	Zanidatamab^a^ (Ziihera)	20 Nov 2024	HER2 (bispecific)	HER2+ biliary tract cancer (unresectable/metastatic, pretreated)	Meric-Bernstam et al. (2022), Harding et al. (2023)
2. Tyrosine Kinase Inhibitors (TKIs)	Repotrectinib^a^	15 Nov 2023	ROS1, ALK, NTRK	ROS1+ NSCLC	Dhillon (2024), Barbato et al. (2024)
	Capivasertib^a^	16 Nov 2023	AKT (PIK3CA/AKT1/PTEN)	HR+/HER2- advanced breast cancer	Oliveira et al. (2024), Rugo et al. (2024), Turner et al. (2023)
	Imatinib	10 May 2001 (capsule) 18 April 2003 (tablet)	BCR-ABL, KIT, PDGFR	CML, GIST	Verweij et al. (2003), Cohen et al. (2002)
	Erlotinib	18 Nov 2004	EGFR	NSCLC (EGFR mutations), pancreatic cancer	Johnson et al. (2005), Shepherd et al. (2004), Burgos Fuster and Sandler (2004), Mo et al. (2007)
	Sunitinib	26 Jan 2006	VEGFR, PDGFR, KIT	RCC, GIST, pancreatic neuroendocrine tumors	Motzer et al. (2007), Raymond et al. (2011), Casali et al. (2006)
	Brigatinib^a^	28 Apr 2017; 2 Oct 2020 (expanded)	ALK	ALK + metastatic NSCLC	Camidge et al. (2018)
	Ripretinib^a^	15 May 2020	KIT, PDGFRα	Advanced GIST after prior therapies	Blay et al. (2020)
	Mobocertinib^a^	15 Sep 2021	EGFR exon 20	NSCLC with EGFR exon 20 insertions (withdrawn October 2023)	Garcia Campelo et al. (2022)
	Futibatinib^a^	30 Sep 2022	FGFR2	FGFR2-altered intrahepatic cholangiocarcinoma	Goyal et al. (2023)
	Osimertinib^a^	16 Feb 2024 (expanded); initial 2015	EGFR	NSCLC with EGFR exon 19 del/L858R (with chemo)	Lu et al. (2024), Soria et al. (2018), Khozin et al. (2017)
3. Immune Checkpoint Inhibitors (ICIs)	Pembrolizumab	4 Sep 2014	PD-1	Melanoma, NSCLC, HNSCC, MSI-H/dMMR tumor-agnostic	Marabelle et al. (2020), Seiwert et al. (2016), Herbst et al. (2016)
	Nivolumab	22 Dec 2014	PD-1	Melanoma, NSCLC, RCC, Hodgkin lymphoma	Rizvi et al. (2015), Weber et al. (2015), Motzer et al. (2015), Herrera et al. (2024)
	Cemiplimab	28 Sep 2018	PD-1	Cutaneous squamous cell carcinoma (CSCC), NSCLC	Migden et al. (2018), Gogishvili et al. (2022)
	Dostarlimab-gxly^a^	9 Feb 2023	PD-1	Recurrent/advanced endometrial cancer (dMMR)	Mirza et al. (2023)
	Tislelizumab^a^	13 Mar 2024; 27 Dec 2024	PD-1	Esophageal SCC; gastric/GEJ adenocarcinoma	Xu et al. (2023), Qiu et al. (2024)
	Atezolizumab	18 May 2016	PD-L1	NSCLC, urothelial carcinoma, TNBC, HCC	Qin et al. (2023), Fehrenbacher et al. (2016), Mittendorf et al. (2020), Rosenberg et al. (2016), Bellmunt et al. (2021), Balar et al. (2017), Galsky et al. (2020)
	Avelumab	23 Mar 2017	PD-L1	Merkel cell carcinoma, urothelial carcinoma, RCC	Kaufman et al. (2016), Patel et al. (2018), Choueiri et al. (2018)
	Durvalumab	1 May 2017	PD-L1	NSCLC, SCLC, biliary tract cancer	Planchard et al. (2016), Antonia et al. (2016), Paz-Ares et al. (2019), Oh et al. (2024)
	Ipilimumab	25 Mar 2011	CTLA-4	Melanoma, NSCLC, RCC, CRC (in combo with nivolumab)	Wolchok et al. (2010), Tykodi et al. (2022), Lenz et al. (2022)
	Tremelimumab^a^	21 Oct 2022	CTLA-4	Unresectable hepatocellular carcinoma (with durvalumab)	Kelley et al. (2021)
4. Antibody-Drug Conjugates (ADCs)	Enhertu^a^ (Fam-trastuzumab deruxtecan-nxki)	5 Aug 2022	HER2-low	Unresectable/metastatic HER2-low breast cancer	Yamashita et al. (2024)
	Sacituzumab govitecan-hziy^a^ (Trodelvy)	22 Apr 2020; 3 Feb 2023	TROP-2	TNBC (2020); HR+/HER2- breast cancer (2023)	Bardia et al. (2017), Rugo et al. (2023)
	Ado-trastuzumab emtansine (Kadcyla)	22 Feb 2013	HER2	HER2+ metastatic breast cancer	Krop et al. (2012)
	Datopotamab deruxtecan^a^ (Datroway)	17 Jan 2025	TROP-2	Unresectable/metastatic HR+/HER2- breast cancer	Royce et al. (2025)
5. CAR-T Cell Therapies	Lisocabtagene maraleucel^a^ (Breyanzi)	24 Jun 2022	CD19	Relapsed/refractory large B cell lymphoma (after 1 therapy)	Makita et al. (2022)
	Idecabtagene vicleucel^a^ (Abecma)	26 Mar 2021	BCMA	Relapsed/refractory multiple myeloma (after 4+ lines)	Munshi et al. (2021)
	Axicabtagene ciloleucel (Yescarta)	18 Oct 2017	CD19	Relapsed/refractory large B cell lymphoma	Neelapu et al. (2017)
	Tisagenlecleucel (Kymriah)	30 Aug 2017	CD19	Relapsed/refractory B cell ALL, large B cell lymphoma	O'Leary et al. (2019)
	Ciltacabtagene autoleucel^a^ (Carvykti)	28 Feb 2022	BCMA	Relapsed/refractory multiple myeloma (after 4+ lines)	Berdeja et al. (2021)

^a^ Denotes recently approved or next-generation therapeutic agents, including drugs with expanded indications, accelerated approval status, or novel molecular designs.

### Epigenetic and mitoepigenetic effects of current cancer therapies

Although conventional and targeted cancer therapies are not designed to directly modify epigenetic machinery, increasing evidence indicates that many of these treatments exert indirect epigenetic and mitoepigenetic effects (Sharma et al., 2010). Chemotherapy and radiotherapy can alter chromatin accessibility and DNA methylation patterns through oxidative stress, while targeted therapies and immune-based treatments reshape cellular metabolism and mitochondrial function, thereby influencing epigenetic states (Reid et al., 2017; Li et al., 2025). Integrating these therapies within an epigenetic framework provides a more comprehensive understanding of treatment response and resistance.

Beyond direct epigenetic targeting, such therapy-induced changes include alterations in DNA methylation patterns, chromatin accessibility, transcriptional reprogramming, and metabolic adaptation driven by mitochondrial stress and redox imbalance (Huang et al., 2012; Martínez-Reyes and Chandel, 2020; Hung et al., 2024). These epigenetically mediated adaptive responses have been implicated in both transient drug tolerance and the emergence of therapy resistance across multiple cancer types. A conceptual summary of these reported interactions between major classes of oncological therapies and epigenetic or mitoepigenetic regulation is provided in Table 2.

**TABLE 2:** Indirect epigenetic and mitoepigenetic effects of contemporary cancer therapies.

Therapy class	Representative drugs	Indirect epigenetic effects	Mitochondrial/Metabolic impact	Relevance to resistance
Chemotherapy	Cisplatin, Doxorubicin	DNA methylation changes, chromatin damage	ROS production, OXPHOS alteration	Epigenetic plasticity
Radiotherapy	–	Chromatin remodeling, histone modifications	Mitochondrial stress, apoptosis	Adaptive transcriptional reprogramming
Targeted therapy	EGFR, BRAF inhibitors	Transcriptional reprogramming	Metabolic rewiring	Drug tolerance
Immunotherapy	PD-1/PD-L1 inhibitors	Epigenetically fixed exhaustion programs	Mitochondrial fitness of T cells	Immune resistance

This table provides a conceptual overview of well-documented indirect epigenetic and mitoepigenetic effects reported across multiple studies and supported by the cited literature, as discussed in the main text.

### Classification and mechanism of actions of epigenetic drugs and compounds

Epigenetic drugs are developed as inhibitors of proteins that add (writers), recognize (readers), and delete (erasers) chemical groups such as methyl and acetyl, to modulate epigenetic modifications. These inhibitors can reverse existing DNA and histone modifications (Table 3) (Suraweera et al., 2025; Kaplánek et al., 2023). Writers are DNA methyltransferase (DNMT), histone acetyltransferase (HAT), and histone methyltransferase (HMT) families. Erasers are histone deacetylases (HDACs), histone demethylases, and the Ten-Eleven Translocation (TET) family.

**TABLE 3:** Epigenetic drugs and compounds.

Epigenetic drug/Compound group	Inhibition of specific enzymes	Name
Drugs/compounds related to the inhibition of histone modifications	HMT inhibitors	Chaetocin (lysine-specific inhibition)
		CPI-1205 (EZH2 inhibitor)
		CPI-169 racemate (EZH2 inhibitor)
		3-Deazaneplanocin A (EZH2 inhibitor)
		EI1 (EZH2 inhibitor)
		EPZ005687 (EZH2 inhibitor)
		EPZ011989 (EZH2 inhibitor)
		GSK126 (EZH2 inhibitor)
		GSK343 (EZH2 inhibitor)
		MAK683 (EED inhibitor 1)
		Pinometostat (DOT1L inhibitor) (EPZ5676)
		Tazemetostat (EZH2 inhibitor)
		Valemetostat (both EZH1 and EZH2 inhibitor)
		ZLD1039 (EZH2 inhibitor)
	HDAC inhibitors	AR-42
		Belinostat
		Bisthianostat
		Chidamide
		Citarinostat
		CUDC-101
		Domatinostat (4SC-202)
		Entinostat
		Fimepinostat
		Givinostat
		Ivaltinostat
		Mocetinostat
		Nanatinostat
		Panobinostat
		Pracinostat
		Psammaplin A
		Quisinostat
		Resminostat
		Ricolinostat
		Romidepsin (Istodax)
		Sodium butyrate
		4-phenylbutyrat
		Sodium valproate
		Sulforaphane
		Tefinostat
		Tinostamustine
		Trichostatin A
		Tucidinostat
		Vorinostat
​	HAT inhibitors	Anacardic acid
		Curcumin
		Hydralazine
		Psammaplin A (PsA)
	Histon demethylase inhibitor	CC-90011
	BET/BRD inhibitors	AZD5153
		BAY1238097
		BET-IN-4 (ODM-207)
		Birabresib (OTX-015, MK-8628)
		BMS-986158
		CPI-0610
		INCB057643
		INCB54329
		JQ1
		Molibresib
		PLX-51107
		(S)-JQ35 (TEN-010)
		Trotabresib
Drugs/compounds related to inhibition of DNA methylation	DNMT inhibitors	Anacardic acid
		5 Azacitidine
		Decitabine
		(−)-Epigallocatechin gallate
		5-Fluoro-2′-deoxycytidine (FdCyd)
		Guadecitabine (S110 or SGI-110)
		Procinamide
		RG108
		DSF (disulfiram)
		SGI-1027
		Zebularine
	DNMT3 inhibitor	Sulforaphane
Drugs/compounds related to inhibition of DNA demethylation	TET inhibitors	Succinate
		Fumarate
		2-Hydroxyglutarate (2-HG)
		Dimethyloxalylglycine
		N-oxalylglycine

**FIGURE 1:**
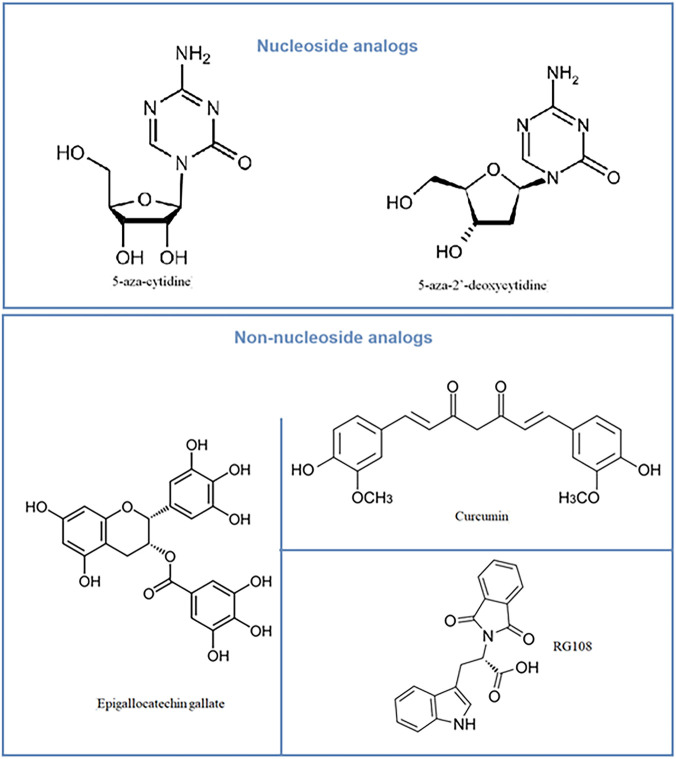
Chemical structures of representative nucleoside and non-nucleoside DNMT inhibitors.

DNMT1 inhibitors include nucleoside analogs or non-nucleoside chemicals. 5′-azacytidine and 5′-deoxy-2′-azacytidine are nucleoside analogs, used as epigenetic drugs due to their similarity to cytosine nucleoside (Suraweera et al., 2025). When these nucleoside analogs are present, they replace methylated cytosines in newly synthesized DNA during DNA replication. Thus, the amount of methylation decreases as the number of methylated cytosines gradually decreases with each replication. Therefore, the epigenetic drug effect of nucleoside analogs is a replication-dependent process. Non-nucleoside analogs contain chemical groups with completely different structures that do not resemble the nucleoside structure (Mohan, 2025). Figure 1 shows some examples of these molecules. Some of these are natural, and some are synthetic compounds. Examples of natural ones are epigallocatechin-3-gallate (Epigallocatechin gallate, EGCG) found in tea and curcumin found in the turmeric plant (Pandey et al., 2025). The most well-known of the synthetic ones is the RG108 molecule. These drugs reduce DNA methylation levels by directly inhibiting the DNMT1 enzyme (Figure 1). Nucleoside analogs 5-azacitidine and 5-aza-2′-deoxy-azacitidine are FDA-approved and are routinely used in cancer treatment.

TET inhibitors are compounds that suppress the activity of TET enzymes—a family of Fe^2+^/α-ketoglutarate–dependent dioxygenases (TET1, TET2, and TET3) responsible for oxidizing 5-methylcytosine (5mC) to 5-hydroxymethylcytosine (5hmC), initiating DNA demethylation. By blocking this process, TET inhibitors lead to DNA hypermethylation, which can alter gene expression patterns, including those involved in differentiation, cell cycle control, and tumor suppression. Common TET inhibitors act by blocking the catalytic activity of TET enzymes, which normally convert 5mC to 5hmC during DNA demethylation. The oncometabolite 2-hydroxyglutarate (2-HG), produced by mutant IDH1/2 enzymes, is a potent competitive inhibitor that mimics α-ketoglutarate and leads to genome-wide DNA hypermethylation. Similarly, dimethyloxalylglycine (DMOG) is a broad-spectrum α-ketoglutarate analog that inhibits TETs and other dioxygenases by occupying their cofactor binding site. Bobcat339 represents a more selective synthetic small-molecule inhibitor that directly targets the TET catalytic domain, reducing 5hmC formation in a dose-dependent manner. Additionally, metabolic intermediates such as succinate and fumarate can accumulate under mitochondrial dysfunction and competitively inhibit α-ketoglutarate-dependent TET activity, linking altered cellular metabolism to epigenetic dysregulation (Kaplánek et al., 2023). Together, these inhibitors modulate DNA methylation landscapes, providing important tools for studying epigenetic regulation and potential therapeutic strategies in cancer and developmental disorders.

Histone acetyltransferase (HAT) inhibitors are divided into three main groups: bisubstrate, natural, and synthetic inhibitors. Lys-CoA p300, one of the bisubstrate inhibitors, binds to the binding site of the HAT enzyme with a specific affinity and inhibits it (Huang et al., 2019). Anacardic acid, curcumin, garcinol, and EGCG, which are natural compounds, have HAT inhibition activity. Studies have shown that synthetic compounds, especially thiazole, isothiazole, and similarly derived compounds with heterocyclic structures, are promising in terms of HAT inhibition (Gorsuch et al., 2009).

The common features of HDAC inhibitors (HDACi) are that they contain three pharmacophore groups, including 1) zinc-binding group (ZBG), 2) linker, and 3) capping group, abbreviated as CAP (Neganova et al., 2022). HDAC inhibitors are often used in combination with other drugs (Smalley et al., 2020). This approach is called the “polypharmacological approach” (Ryszkiewicz et al., 2025). Polypharmacology is defined as the design or use of pharmaceutical agents that act on more than one target. Pharmaceutical agents can be applied as combinations of multiple drugs that bind to different targets; this approach is called drug combination, or can be a single drug that binds to various targets, defined as multi-target ligands (Lembo and Bottegoni, 2024).

The bromodomain is a protein region of approximately 110 amino acids that recognizes acetylated lysines at the N-terminal ends of histones. Proteins containing this region are known as the BET (bromodomain and extra-terminal motif-containing) protein family. The mammalian BET protein family has four members: BRD2, BRD3, BRD4, and BRDT, and these proteins are associated with cancer and immunity (To et al., 2023). The most well-known BET inhibitor is the JQ1 molecule, which inhibits BRD4. BRD4 is a type of epigenetic reader protein and is involved in histone acetylation, gene transcription, and alternative splicing mechanisms (Zhang et al., 2021; Zhou et al., 2020). BRD4 protein binds to the acetylated lysine residues of histones and reads them. This reading directs the RNA polymerase II towards transcription. Thus, it contributes to gene expression in conjunction with histone acetylation. When the JQ1 molecule inhibits BRD4, active transcription cannot occur (Dey et al., 2003).

The most studied group of HMT inhibitors is EZH2 inhibitors. EZH2 is a type of HMT enzyme and is involved in maintaining the heterochromatin structure. Since it has been found that the EZH2 gene, which encodes the EZH2 enzyme, is generally overexpressed in cancer, inhibition of this enzyme is one of the targeted mechanisms for cancer treatment. The drug Valemetostat has the potential to inhibit both EZH1 and EZH2 activity. Since both enzymes show increased activity in some types of cancer, Valemetostat harbors promising potential against these cancers (Liu and Yang, 2023). Pinometostat is the first identified HMT inhibitor that has reached the Phase 1 clinical stage for use in the treatment of leukemia (Menghrajani et al., 2019).

Among the histone demethylases, most studies are on the lysine-specific histone demethylase 1A (LSD1) enzyme. LSD1 enzyme is an enzyme that specifically removes methyl groups from H3K4me1/2 and H3K9me1/2 modifications. In many cancer cells, histone demethylase enzymes such as LSD1 show more activity than in normal cells. LSD1 inhibitors are divided into two groups: covalent and non-covalent. Each group includes some hybrid compounds (dual or multi-target compounds) that can simultaneously inhibit LSD1 and other targets. To date, 9 LSD1 inhibitors for hematological and/or solid cancers have entered clinical trials (Noce et al., 2023).

### The use of standard epigenetic drugs in cancer therapies

Epigenetic modifications play crucial roles in cancer progression by regulating gene expression without altering the DNA sequence. Epigenetic drugs primarily target DNA methylation, histone modification, and chromatin remodeling enzymes. These include DNMTs, HDACs, and BRDs of BET proteins (Suraweera et al., 2025). Epigenetic therapies have revolutionized the treatment of hematologic malignancies and lymphomas by targeting aberrant epigenetic modifications, such as DNA hypermethylation and histone deacetylation (Olsen et al., 2007; Piekarz et al., 2011). These modifications often silence tumor suppressor genes, contributing to cancer progression. This review synthesizes the clinical development, mechanisms of action, and efficacy of five epigenetic drugs approved by the FDA before 2015: Azacitidine, Decitabine, Vorinostat, Romidepsin, and Belinostat. These agents target DNMTs or HDACs, offering novel therapeutic alternatives for myelodysplastic syndromes (MDS), acute myeloid leukemia (AML), cutaneous T cell lymphoma (CTCL), peripheral T cell lymphoma (PTCL), and multiple myeloma.

### DNA methyltransferase inhibitors (DNMTi)

#### Azacitidine and decitabine

Azacitidine is a pyrimidine nucleoside analog that inhibits DNMT1, resulting in DNA hypomethylation and reactivation of silenced genes (Kagan et al., 2023). Fenaux et al. (2009) conducted a phase III trial in 358 higher-risk MDS patients, showing that azacitidine significantly improved median overall survival (24.5 vs. 15.0 months) compared with conventional care, establishing it as the first therapy to extend survival in MDS (Fenaux et al., 2009). Decitabine is another DNMT1 inhibitor with a mechanism similar to Azacitidine (Kagan et al., 2023). Kantarjian et al. (2006) conducted a phase III trial in MDS, showing that decitabine produced durable clinical responses, improved hematologic function, and delayed progression to AML or death, confirming its therapeutic value in higher-risk MDS (Kantarjian et al., 2006). However, the study showed the ability of Decitabine to alter disease course through epigenetic modulation, though survival benefits were less pronounced than with Azacitidine.

### Histone deacetylase inhibitors (HDACi)

#### Vorinostat

Duvic et al. (2007) evaluated vorinostat, an HDACi, in 33 refractory CTCL patients, achieving a 24% response rate with manageable toxicity; the 400 mg daily dose was best tolerated (Duvic et al., 2007). Olsen et al. (2007) conducted a phase IIb trial of vorinostat (400 mg daily) in 74 refractory CTCL patients, achieving a 29.7% response rate and a median response duration of ≥185 days (Olsen et al., 2007).

#### Romidepsin

Romidepsin, as a class I HDACi, received FDA approval for CTCL and PTCL. Piekarz et al. (2009) evaluated romidepsin monotherapy in 71 CTCL patients, reporting a 34% overall response rate (4 complete, 20 partial responses) and a median response duration of 13.7 months (Piekarz et al., 2009). Later, Piekarz et al. (2011) evaluated romidepsin in 47 relapsed PTCL patients, showing a 38% response rate with a median response duration of 8.9 months (Piekarz et al., 2011).

#### Belinostat

Lee et al. (2015) detailed the FDA approval process for belinostat, a histone deacetylase inhibitor approved for relapsed or refractory PTCL. The approval was based on a single-arm phase II trial in 120 evaluable patients, showing a 25.8% overall response rate (10.8% complete, 15.0% partial) and a median duration of response of 8.4 months (Lee et al., 2015). Poole, (2014) further reviewed the development and first global approval of belinostat, a hydroxamate-type inhibitor of class I, II, and IV HDACs, highlighting its accelerated FDA approval as monotherapy for relapsed or refractory PTCL (Poole, 2014).

**FIGURE 2:**
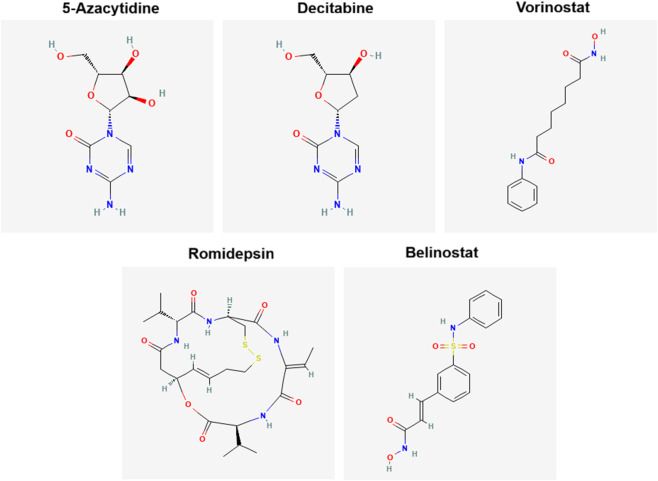
Chemical structures of standard epigenetic therapeutics (obtained by PubChem).

The pre-2015 epigenetic therapies (Azacitidine, Decitabine, Vorinostat, Romidepsin, and Belinostat) represent a paradigm shift in cancer treatment by targeting epigenetic dysregulation (Table 3). DNMT inhibitors, Azacitidine and Decitabine, have proven transformative in MDS and AML, improving survival and delaying disease progression. HDAC inhibitors (Vorinostat, Romidepsin, and Belinostat) have expanded options for CTCL and PTCL, particularly in relapsed/refractory settings. Figure 2 illustrates the 2D chemical structures of standard epigenetic therapeutics given in Table 4.

**TABLE 4:** Standard epigenetic therapeutics.

Drug name	FDA approval date	Target	Use	References
Azacitidine	19 May 2004	DNMT1	MDS, AML	Fenaux et al. (2009), Issa et al. (2004)
Decitabine	2 May 2006	DNMT1	MDS, AML	Kantarjian et al. (2006)
Vorinostat	6 Oct 2006	HDAC	CTCL	Olsen et al. (2007), Duvic et al. (2007)
Romidepsin	5 Nov 2009 (CTCL); 17 Jun 2011 (PTCL)	HDAC, Class I	CTCL, PTCL	Piekarz et al. (2011), Piekarz et al. (2009)
Belinostat	3 Jul 2014	HDAC	Relapsed/refractory PTCL	Lee et al. (2015), Poole, (2014)

## Novel epigenetic therapies

The landscape of epigenetic therapies has evolved significantly since 2015, with novel agents (approved 2020-2025 or in Development) targeting specific epigenetic regulators beyond traditional DNMTs and HDACs. This review focuses on seven innovative epigenetic therapies approved between 2020 and 2025 or currently in development: Enasidenib, Ivosidenib, Tazemetostat, Pinometostat (EPZ-5676), Chidamide, Entinostat, and Pracinostat (Table 4). These drugs target isocitrate dehydrogenases (IDH1/2), histone methyltransferases (EZH2, DOT1L), and HDACs, offering precision approaches for AML, cholangiocarcinoma, lymphomas, and breast cancer. While some have gained regulatory approval, others remain in clinical trials, reflecting ongoing efforts to expand epigenetic therapeutic options.

### IDH inhibitors

#### Enasidenib

Enasidenib targets mutant IDH2, an enzyme that produces the oncometabolite 2-HG, leading to histone and DNA hypermethylation. Stein et al. (2017) conducted a phase I/II first-in-human trial of enasidenib (AG-221), a selective oral inhibitor of mutant IDH2, in patients with advanced myeloid malignancies, primarily relapsed or refractory AML. Among patients with mutant IDH2 AML, enasidenib 100 mg daily induced an overall response rate of 40.3% and a median response duration of 5.8 months, with complete remission in 19% of patients (Stein et al., 2017). Thus, it could be mechanistically proposed that enasidenib blocks the production of the oncometabolite 2-hydroxyglutarate, reversing DNA and histone hypermethylation and promoting myeloid differentiation rather than cytotoxicity, establishing a new epigenetic differentiation-based therapy for IDH2-mutant AML.

#### Ivosidenib

Ivosidenib, an IDH1 inhibitor, received FDA approval for relapsed/refractory AML and IDH1-mutated cholangiocarcinoma. DiNardo et al. (2018) conducted a phase I dose-escalation and expansion study of ivosidenib (AG-120), an oral selective inhibitor of mutant IDH1, in IDH1-mutated relapsed or refractory AML. Among 179 patients, ivosidenib induced an overall response rate of 41.6%, with complete remission (CR) or CR with partial hematologic recovery in 30.4% and median response durations of 6–9 months (DiNardo et al., 2018). Later, Abou-Alfa et al. (2020) conducted the ClarIDHy phase III trial showing that ivosidenib, an IDH1 inhibitor, significantly improved progression-free survival and was well tolerated in patients with previously treated IDH1-mutant cholangiocarcinoma, marking the first targeted therapy to show benefit in this disease (Abou-Alfa et al., 2020).

### Histone methyltransferase inhibitors (HMTi)

#### Tazemetostat

Tazemetostat inhibits EZH2, an HMT responsible for H3K27 trimethylation, often dysregulated in lymphomas (Julia and Salles, 2021). Morschhauser et al. (2020) showed that tazemetostat, an EZH2 inhibitor, produced durable responses in relapsed or refractory follicular lymphoma, with higher efficacy in EZH2-mutant (69%) than wild-type (35%) cases and a favorable safety profile (Morschhauser et al., 2020).

#### Pinometostat (EPZ-5676)

Pinometostat, a DOT1L inhibitor, targets histone H3K79 methylation and remains in phase I/II development for MLL-rearranged acute leukemia (Stein et al., 2018). Stein et al. (2018) conducted a phase I clinical trial evaluating pinometostat (EPZ-5676), a first-in-class DOT1L inhibitor, in 51 adults with advanced acute leukemias, particularly those with MLL gene rearrangements (MLL-r). The drug was administered as a continuous IV infusion in 28-day cycles across multiple dose levels. Pinometostat reduced H3K79 methylation, confirming target engagement. Two patients with t(11; 19) achieved complete remission, demonstrating proof of concept for DOT1L inhibition in MLL-r leukemia, though overall clinical activity was modest as monotherapy (Stein et al., 2018).

### Histone deacetylase inhibitors (HDACi)

#### Chidamide

Chidamide is a class I HDACi for relapsed/refractory PTCL (Lu et al., 2016). Shi et al. (2017) conducted a multicenter real-world study in 383 patients with relapsed or refractory PTCL to evaluate chidamide, a subtype-selective class I HDACi. In monotherapy, the overall response rate (ORR) was 39%, and in combination with chemotherapy (Shi et al., 2017).

#### Entinostat

Entinostat, a class I HDACi, is in phase III trials for hormone receptor-positive (HR+) advanced breast cancer in combination with exemestane (Wang et al., 2024). Yeruva et al. (2018) described the E2112 phase III trial, designed to evaluate entinostat, a histone deacetylase (HDAC) inhibitor, in combination with exemestane *versus* placebo plus exemestane in hormone receptor–positive advanced breast cancer resistant to non-steroidal aromatase inhibitors. The study builds on prior phase II ENCORE 301 results, which showed improved progression-free and overall survival with entinostat. E2112 aims to confirm the role of HDAC inhibition in overcoming endocrine resistance through epigenetic modulation in advanced breast cancer (Yeruva et al., 2018; Connolly et al., 2021).

#### Pracinostat

Pracinostat, a pan-HDACi, is in phase III trials for AML and MDS (Dai et al., 2024). Garcia-Manero et al. (2024) conducted the PRIMULA phase III trial evaluating pracinostat, an oral pan-HDACi, combined with azacitidine *versus* azacitidine alone in newly diagnosed AML patients ineligible for intensive chemotherapy. Among 406 randomized patients, no improvement in overall survival was observed (median 9.95 months in both groups; *p* = 0.8275), and secondary endpoints showed no clinical benefit. The addition of pracinostat to azacitidine did not improve outcomes in elderly or unfit AML patients, despite a strong preclinical rationale for combining HDAC and DNA hypomethylating agents (Garcia-Manero et al., 2024). Earlier, Yalniz et al. (2020) conducted a phase II trial evaluating the addition of pracinostat, a pan-HDACi, to hypomethylating agents in MDS patients who had not responded to prior hypomethylating agent therapy. Among 45 patients, the clinical improvement rate was low, with frequent grade ≥3 toxicities leading to treatment discontinuation. The combination did not enhance outcomes in hypomethylating agent-refractory myelodysplastic syndromes, likely due to toxicity-related dose reductions and suboptimal exposure (Yalniz et al., 2020).

**FIGURE 3:**
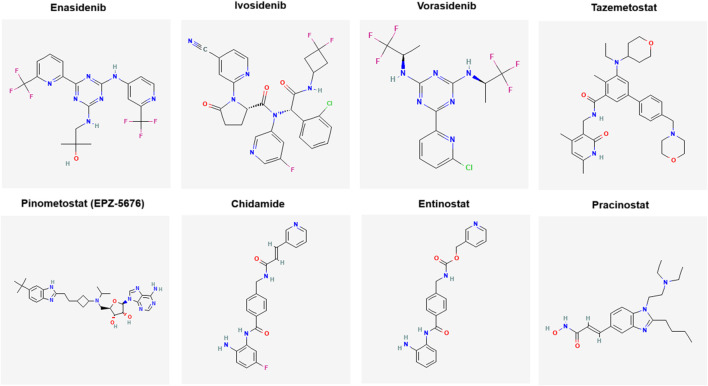
Chemical structures of novel epigenetic therapeutics (obtained by PubChem).

Novel epigenetic therapies approved between 2020 and 2025 (*e.g.*, Tazemetostat) or in development (*e.g.*, Entinostat, Pracinostat, Pinometostat) expand the therapeutic arsenal beyond traditional DNMT and HDAC inhibitors (Table 5). IDH inhibitors (Enasidenib, Ivosidenib) have solidified precision medicine in AML and cholangiocarcinoma, through mutation-specific epigenetic targeting. The success of Tazemetostat in follicular lymphoma shows the potential of HMT inhibitors, while the regional adoption of Chidamide reflects global inequalities in drug access. However, agents like Pracinostat and Pinometostat face difficulties, with limited efficacy or toxicity concerns slowing progress. These therapies show both the therapeutic potential and the unresolved complexity of targeting epigenetic mechanisms, especially in solid tumors, where efficacy remains inconsistent and biomarkers are still evolving. Figure 3 illustrates the 2-D chemical structures of novel epigenetic therapeutics given in Table 5.

**TABLE 5:** Novel epigenetic therapeutics.

Drug name	FDA approval date	Target	Use	References
Enasidenib	1 Aug 2017	IDH2	Relapsed/refractory AML with IDH2 mutation	Stein et al. (2017)
Ivosidenib	20 Jul 2018 (AML); 25 May 2022 (cholangiocarcinoma)	IDH1	Relapsed/refractory AML; IDH1-mutated cholangiocarcinoma	DiNardo et al. (2018), Abou-Alfa et al. (2020), Zhu et al. (2021)
Vorasidenib	6 Aug 2024	IDH1/2	IDH mutated low grade gliomas	Mellinghoff et al. (2023a), Mellinghoff et al. (2023b)
Tazemetostat	18 Jun 2020	EZH2 (HMT)	Relapsed/refractory follicular lymphoma (EZH2 mutation)	Morschhauser et al. (2020)
Pinometostat (EPZ-5676)	Not FDA-approved (Phase I/II ongoing)	DOT1L (HMT)	MLL-rearranged acute leukemia (trials ongoing)	Stein et al. (2018)
Chidamide	Not FDA-approved (China: December 2014)	HDAC (Class I)	Relapsed/refractory PTCL (approved in China)	Shi et al. (2017)
Entinostat	Not FDA-approved (Phase III ongoing)	HDAC (Class I)	HR + advanced breast cancer (with exemestane, trials ongoing)	Yeruva et al. (2018), Connolly et al. (2021)
Pracinostat	Not FDA-approved (Phase III ongoing)	HDAC (Pan)	AML, MDS (trials ongoing)	Garcia-Manero et al. (2024), Yalniz et al. (2020)

Epigenetic therapies demonstrate a dynamic interplay of innovation and obstacles, as evidenced by the progression from standard pre-2015 drugs like Azacitidine and Vorinostat to novel agents approved or in development between 2020 and 2025, such as Vorasidenib and Tazemetostat. A prominent trend is the rise of precision-targeted therapies, with drugs like Vorasidenib, approved in 2024 for IDH1/2-mutated low-grade gliomas (Mellinghoff et al., 2023a), and Tazemetostat, approved in 2020 for EZH2-mutated follicular lymphoma (Morschhauser et al., 2020), demonstrating mutation-specific efficacy. Combination strategies are also advancing with ongoing trials exploring pairings such as Pracinostat with Azacitidine in AML (Garcia-Manero et al., 2024). The field is expanding beyond hematologic cancers into solid tumors and non-oncologic applications, as evidenced by the approval of Ivosidenib for cholangiocarcinoma (Abou-Alfa et al., 2020) and preclinical exploration of HDAC inhibitors such as Chidamide (Shi et al., 2017). However, significant challenges still exist; efficacy in solid tumors remains inconsistent, with drugs like Entinostat providing limited benefit in breast cancer. (Connolly et al., 2021), echoing earlier struggles with Vorinostat in CTCL (Duvic et al., 2007). Toxicity persists, as broad-acting agents like Belinostat (Lee et al., 2015) and Pinometostat (Stein et al., 2018) exhibit dose-limiting side effects, while resistance to DNMT inhibitors, such as Decitabine, complicates long-term use (Kantarjian et al., 2006). Translational and regulatory difficulties further delay progress, with agents such as Pracinostat still awaiting approval despite extensive study (Yalniz et al., 2020), showing the need for refined strategies to utilize the potential of epigenetic therapies.

A significant trend is the development of precision epigenetic therapies tailored to specific molecular alterations. Vorasidenib, approved in 2024 for IDH1/2-mutated low-grade gliomas (Mellinghoff et al., 2023b), and Tazemetostat, targeting EZH2 mutations in follicular lymphoma (Morschhauser et al., 2020), exemplify this shift toward mutation-specific treatments, improving outcomes over broader-acting agents like Vorinostat (Duvic et al., 2007). This precision enhances therapeutic efficacy in genetically defined subsets of cancer.

Ongoing trials, including the combination of Pracinostat with Azacitidine in AML (Garcia-Manero et al., 2024), illustrate current efforts to enhance therapeutic efficacy through epigenetic combination strategies. While these approaches primarily target nuclear epigenetic regulators, recent preclinical and translational studies suggest that incorporating mitochondrial apoptosis and metabolic vulnerabilities may further potentiate therapeutic responses in selected hematologic malignancies. A recent preclinical study demonstrated that the HDAC inhibitor panobinostat significantly sensitizes AraC-resistant AML cells to the azacitidine–venetoclax combination by suppressing c-Myc signaling and altering mitochondrial metabolism, including oxidative phosphorylation and glycolysis, thereby enhancing apoptosis *in vitro* (Zhao et al., 2024). Consistent with these findings, recent preclinical and clinical case-series data in T cell acute lymphoblastic leukemia reported synergistic anti-proliferative effects of a triplet regimen combining azacitidine, the HDAC inhibitor chidamide, and venetoclax, with encouraging remission outcomes observed in a small cohort of high-risk patients (Zheng et al., 2025a).

The field advances with next-generation epigenetic drugs designed for improved specificity and reduced toxicity. Pinometostat, targeting DOT1L in MLL-rearranged leukemia (Stein et al., 2018), and emerging HDAC inhibitors like Pracinostat (Yalniz et al., 2020) represent efforts to refine earlier drugs like Belinostat (Lee et al., 2015). These developments reflect ongoing efforts to improve the clinical relevance of epigenetic therapies, particularly in cancers that have been difficult to treat with existing agents.

Biomarker-driven strategies are increasingly being adopted to refine patient stratification and improve treatment outcomes. EZH2 mutation status guides Tazemetostat use (Morschhauser et al., 2020), while IDH mutations direct Vorasidenib and Ivosidenib therapies (Abou-Alfa et al., 2020; Mellinghoff et al., 2023b). Building on earlier trials with Decitabine (Kantarjian et al., 2006), this trend reflects a growing effort to tailor cancer therapies to individual patient profiles, though achieving consistently improved response rates remains a complex challenge.

Drug resistance continues to challenge long-term efficacy. The therapeutic benefits of Decitabine in MDS decrease over time due to adaptive resistance (Kantarjian et al., 2006), and similar issues are emerging with EZH2 inhibitors such as Tazemetostat (Morschhauser et al., 2020). This necessitates novel strategies to sustain therapeutic impact. Translational challenges remain significant, as encouraging preclinical findings do not always translate into clinical success. Pinometostat reduced H3K79 methylation but showed modest activity in leukemia patients (Stein et al., 2018), echoing difficulties seen with earlier agents like Romidepsin (Piekarz et al., 2011). These gaps emphasize the need for better predictive models.

## The role of mitoepigenetics in cancer with *in vitro* and *in vivo* insights on mitochondrial regulation and cancer progression

**FIGURE 4:**
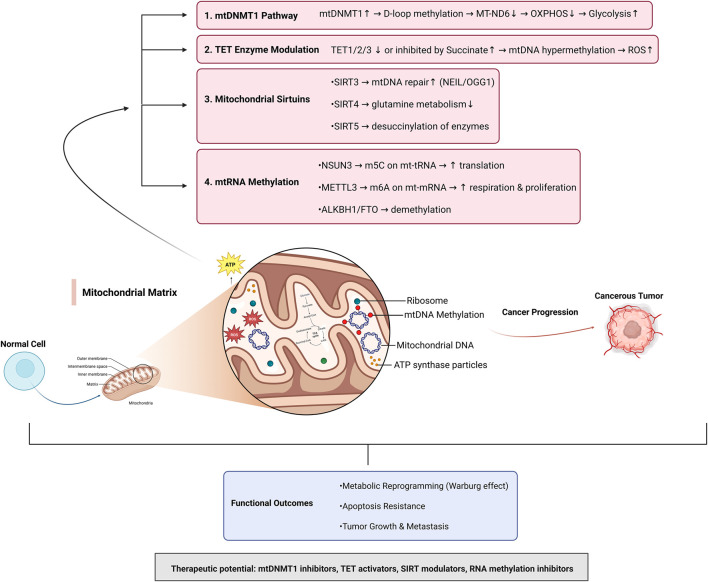
Integrated Mitoepigenetic Mechanisms Linking Mitochondrial Function to Cancer Progression. The pathways shown in this integrative model are derived from peer-reviewed *in vitro* and/or *in vivo* cancer studies addressing mtDNA methylation, mitochondrial sirtuin activity, RNA methylation, and metabolic reprogramming. Supporting references for individual signaling axes are provided in the main text.

Mitoepigenetics encompasses the study of epigenetic mechanisms that regulate the mitochondrial genome (mtDNA) and its associated proteins. Mitochondria, essential for cellular energy production and signaling, are particularly susceptible to damage due to their proximity to reactive oxygen species (ROS) generated during oxidative phosphorylation (OXPHOS) (Coppedè and Stoccoro, 2019). Unlike nuclear DNA, mtDNA is compact, circular, and lacks robust protective structures such as histones, rendering it vulnerable to oxidative stress-induced damage and epigenetic alterations. These alterations include DNA methylation, regulation by non-coding RNAs, and modifications of mitochondrial histone-like proteins (Stoccoro and Coppedè, 2021). Such epigenetic changes can disrupt mitochondrial function, contributing to altered cellular metabolism and signaling pathways. In cancer, the mitochondrial epigenome undergoes significant reprogramming to support tumor development and progression. Mitochondria are reprogrammed to enhance metabolic flexibility, evade apoptosis, and enable metastasis. This reprogramming involves epigenetic alterations in mtDNA that regulate mitochondrial gene expression, leading to changes in energy production and metabolic pathways (Dong et al., 2020). One key feature of this reprogramming is the shift from OXPHOS to aerobic glycolysis, commonly referred to as the Warburg effect. This shift allows cancer cells to meet their increased energy demands while producing biosynthetic precursors required for rapid proliferation. Moreover, mtDNA modifications such as hypermethylation of the D-loop region-a critical control site for mtDNA replication and transcription—have been implicated in tumor aggressiveness and metastasis (Yue et al., 2022). Additionally, non-coding RNAs, including microRNAs and long non-coding RNAs, play essential roles in mitochondrial-nuclear communication, influencing cancer cell survival and immune evasion (Mosca et al., 2021). These findings illustrate the role of mitoepigenetics in linking mitochondrial dysfunction with oncogenic signaling pathways (Sun et al., 2018). By altering mitochondrial energy metabolism and signaling, mitoepigenetic modifications contribute to hallmark processes of cancer, including sustained proliferation, resistance to apoptosis, and enhanced metastatic potential (Sumiyoshi et al., 2022). As such, understanding mitoepigenetics dynamics provides valuable insights into tumor biology and presents novel therapeutic targets for cancer treatment (Figure 4). The mtDNMT1 pathway enhances D-loop methylation, resulting in alterations in mitochondrial transcription and a metabolic shift from OXPHOS to glycolysis, a characteristic of the Warburg effect. TET enzyme modulation affects mtDNA demethylation; when inhibited by metabolites such as succinate, TET enzymes promote mtDNA hypermethylation and the accumulation of reactive oxygen species (ROS). Mitochondrial sirtuins (SIRT3, SIRT4, SIRT5) regulate key processes such as mtDNA repair, glutamine metabolism, and enzyme desuccinylation, maintaining mitochondrial integrity. mtRNA methylation, mediated by NSUN3 and METTL3, enhances mitochondrial translation, respiration, and proliferation, while ALKBH1 and FTO function as demethylases. Together, these mechanisms drive metabolic reprogramming, apoptosis resistance, and tumor growth and metastasis (Figure 4). The interactions presented in Figure 4 reflect experimentally supported mechanisms reported across cancer cell line studies, xenograft models, and genetically engineered systems. Thus, potential therapeutic strategies targeting mitoepigenetic dynamics may cover mtDNMT1 inhibitors, TET activators, SIRT modulators, and RNA methylation inhibitors, to restore mitochondrial epigenetic balance and counteract cancer progression.

While current FDA-approved epigenetic drugs primarily target nuclear DNA methylation, histone modifications, or related nuclear epigenetic alterations, their effects on mtDNA are indirect and incidental. These drugs influence cellular metabolism—sometimes impacting mitochondrial function via nuclear-encoded genes, such as *PGC-1α*, or downstream metabolic shifts—but they are not designed to target mtDNA epigenetics directly (Abu Shelbayeh et al., 2023). However, the growing recognition of mtDNA epigenetic modifications in cancer and their role in mitochondrial metabolism has driven preclinical research, particularly *in vitro*, into novel pathways that could lead to mtDNA-specific epigenetic therapies.

### Mitochondrial DNMT1 (mtDNMT1) inhibition

Mitochondrial DNMT1 (mtDNMT1), a form of DNMT1 that localizes to mitochondria, methylates mtDNA, regulating genes like *MT-ND6*, which is vital for oxidative phosphorylation. Altered mtDNA methylation is observed in cancer cells (*e.g.*, colorectal cancer) and may affect mitochondrial metabolism, with implications for cancer biology (Shock et al., 2011). Experiments in cancer cell lines (*e.g.*, HCT116) using mtDNMT1 knockdown demonstrate reduced mtDNA methylation, increasing transcription of genes like *MT-ND6*, though the specificity relative to nuclear DNA methylation was not assessed in this study (Shock et al., 2011). No clinical trials explicitly target mtDNMT1 currently. Developing mtDNMT1-specific inhibitors could offer a novel cancer therapy by directly disrupting mitochondrial bioenergetics.

### Mitochondrial TET enzyme modulation

Mitochondrial TET enzyme modulation is a promising area in mitoepigenetics, potentially impacting cancer by altering mtDNA demethylation and mitochondrial function. TET enzymes (TET1, TET2, TET3) oxidize 5mC to 5hmC to promote DNA demethylation. These enzymes may function in mitochondria, where mtDNA 5hmC could regulate gene expression critical to cancer metabolism, such as oxidative phosphorylation and ROS production (Kaplánek et al., 2023). mtDNA epigenetic changes, including 5hmC dysregulation, are observed in cancers like leukemia and hepatocellular carcinoma, suggesting the crucial roles of mitochondrial TETs in tumor progression (Kaplánek et al., 2023). This concept is indirectly supported by Cramer-Morales et al. (2020), who demonstrated that succinate accumulation, caused by mitochondrial MnSOD depletion, inhibits TET activity, leading to nuclear DNA hypermethylation and altered cell fate in erythroleukemia models (*e.g.*, HEL 92.1.7 cells). While their focus was nuclear, their finding suggested that succinate, a TET inhibitor, disrupts epigenetic regulation, suggesting a potential parallel effect on mitochondrial TET activity, which could influence mtDNA methylation and mitochondrial function in cancer cells under oxidative stress (Cramer-Morales et al., 2020), as (Kaplánek et al., 2023) suggests for broader mitoepigenetic dynamics. However, direct evidence of mitochondrial TET activity in cancer remains limited, requiring further research. Enhancing or inhibiting mitochondrial TET activity could offer a new epigenetic mechanism for mtDNA regulation in cancer.

### Mitochondrial sirtuin (SIRT3/4/5) modulation

The interplay between SIRT3, SIRT4, and SIRT5 in coordinating mitochondrial responses shows their collective impact on mitoepigenetic regulation. SIRT4, known for its role in repressing glutamine metabolism, may inhibit cancer cell proliferation by limiting nutrient availability, though specific studies on its modulation in cancer remain sparse. Jeong et al. (2013) demonstrated that SIRT4 inhibits mitochondrial glutamine metabolism by ADP-ribosylating and repressing glutamate dehydrogenase, reducing glutamine flux into the tricarboxylic acid cycle, and this tumor-suppressive activity was linked to decreased proliferation in cancer cells (Jeong et al., 2013). Similarly, SIRT5, which regulates lysine desuccinylation and demalonylation, fine-tunes mitochondrial enzyme activity. Du et al. (2011) established SIRT5 as an NAD + -dependent lysine demalonylase and desuccinylase, showing its ability to remove these modifications from mitochondrial proteins (Du et al., 2011), while Greene et al. (2019) found that SIRT5 stabilizes glutaminase via desuccinylation in breast cancer cells, enhancing glutamine metabolism and potentially supporting tumor growth or survival under stress conditions (Greene et al., 2019). Lei et al. (2024) emphasize that mitochondrial transcription, modulated by sirtuins, influences cancer cell survival and proliferation. Specifically, SIRT3 regulates mitochondrial biogenesis and metabolism, which are often dysregulated in cancer cells to support rapid growth and adaptation to stress (Lei et al., 2024). In colon cancer, Torrens-Mas et al. (2019) demonstrated that silencing SIRT3 impairs mitochondrial biogenesis and disrupts metabolic homeostasis, leading to reduced cell viability and increased oxidative stress *in vitro* (Torrens-Mas et al., 2019). These findings suggest that SIRT3 may function as a metabolic gatekeeper; however, its role appears context-dependent and dualistic, with studies indicating both tumor-promoting and tumor-suppressive activities depending on the cancer type and stage. Its tumor-suppressive role is also evident, as SIRT3 enhances mitochondrial integrity, which may counteract the Warburg effect. Beyond metabolism, SIRT3 modulates mtDNA repair, a critical mechanism for maintaining genomic stability in cancer cells. Kabziński et al. (2019) revealed that in colorectal cancer, SIRT3 deacetylates key mtDNA repair enzymes, including NEIL1, NEIL2, OGG1, MUTYH, APE1, and LIG3. This deacetylation enhances their activity, reducing mtDNA damage accumulation, which is a factor linked to cancer progression and chemotherapy resistance (Kabziński et al., 2019). The study suggests that the regulation of mtDNA repair by SIRT3 could serve as a therapeutic target, as its inhibition might sensitize cancer cells to oxidative damage and apoptosis. While SIRT3 has garnered significant attention, SIRT4 and SIRT5 also contribute to mitochondrial regulation in cancer, albeit with less characterized mechanisms. Lei et al. (2024) note that mitochondrial sirtuins influence transcription of mtDNA-encoded genes, which are essential for respiratory chain function (Lei et al., 2024). TTorrens-Mas et al. (2019) suggest that SIRT3 silencing in animal models of colon cancer could disrupt tumor progression by compromising mitochondrial function, aligning with *in vitro* observations (Torrens-Mas et al., 2019). Similarly, Kabziński et al. (2019) propose that targeting SIRT3-mediated mtDNA repair pathways in colorectal cancer could enhance therapeutic efficacy, offering a translational bridge from bench to bedside (Kabziński et al., 2019). The modulation of mitochondrial sirtuins (SIRT3/4/5) thus emerges as a pivotal factor in cancer biology. The dual role of SIRT3 in metabolism and mtDNA repair, alongside the emerging functions of SIRT4 and SIRT5, shows their potential as therapeutic targets. Future research should focus on elucidating the context-specific roles of SIRT4 and SIRT5 and exploring combinatorial strategies to manipulate mitochondrial sirtuin activity, offering new avenues for combating cancer through mitoepigenetic intervention.

### Mitochondrial RNA methylation (m^5^C/m^6^A writers and erasers)

Mitochondrial RNA (mtRNA) methylation, including m^5^C and m^6^A modifications, is a critical mitoepigenetic process in cancer, driven by writers and potentially erasers that regulate mitochondrial function and tumor progression. NSUN3, an m^5^C writer, methylates mitochondrial tRNA-Met at cytosine 34 (m^5^C34), enhancing translation of mtDNA-encoded proteins vital for oxidative phosphorylation (Van Haute et al., 2016). Van Haute et al. (2016) demonstrated that NSUN3 deficiency impairs this methylation, reducing mitochondrial protein synthesis in patient-derived cells. mtRNA methylation, particularly m5C modification, plays a critical role in regulating mitochondrial gene expression and function. NSUN family methyltransferases, including NSUN2, NSUN3, and NSUN4, act as key m5C writers within mitochondria (Li et al., 2022). NSUN3 primarily modifies mitochondrial tRNAs, while NSUN4 targets 12S rRNA, contributing to mitoribosome assembly and mitochondrial translation. Additionally, the demethylase ALKBH1 functions as an m5C eraser by oxidizing m5C to 5-formylcytosine (f5C) in mitochondrial tRNAs, influencing RNA stability. Dysregulation of these enzymes may alter mitochondrial metabolism and contribute to cancer progression by impacting cellular energy production and apoptosis pathways (Li et al., 2022).

mtRNA methylation, particularly N6-methyladenosine (m6A), plays a pivotal role in regulating mitochondrial function and cancer progression. METTL3, an m6A methyltransferase, has been shown to methylate mtRNAs, thereby enhancing mitochondrial respiration. In colorectal cancer cells, METTL3 installs m6A modifications on mitochondrial mRNAs and rRNAs, leading to increased proliferation *in vitro* and tumor growth *in vivo* (Pan et al., 2022).

**FIGURE 5:**
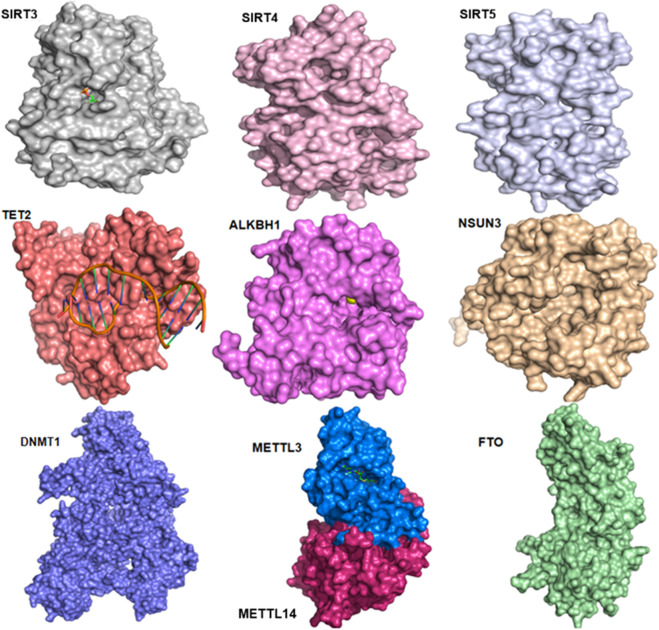
AlphaFold 3-based modeling or X-ray crystallography-based 3D structures of proteins operate essential roles during mitoepigenetic regulations. SIRT3 (PDB ID: 4BN4), SIRT4 (Model ID: AF-Q9Y6E7-F1), SIRT5 (Model ID: AF-Q5R6G3-F1), TET2 (PDB ID: 5DEU), ALKBH1 (PDB ID: 6, IE3), NSUN3 (Model ID: AF-Q9H649-F1), DNMT1 (PDB ID: 4WXX), METTL3 and 14 (PDB ID: 5IL2), and FTO (PDB ID: 4IE5).

Consequently, considering their fundamental and differentiated roles in mitochondrial processes, the three-dimensional (3D) structures of the studied mitoepigenetic proteins are exemplified here to further highlight their differences (Figure 5).

Regarding m6A demethylases, ALKBH5 is known to demethylate nuclear m6A RNAs. ALKBH5 suppresses gastric cancer progression by demethylating m6A on uncapped WRAP53 RNA isoforms, reducing their translation and impacting downstream pathways. ALKBH5 downregulation is associated with poor prognosis, supporting its tumor-suppressive role in gastric cancer (Zheng et al., 2025b). Zeng et al. (2024) suggest that FTO may demethylate mitochondrial m6A, though its specific role in cancer is still under exploration Zeng et al. (2024). Dysregulation of mtRNA methylation by enzymes such as NSUN3 and METTL3 can alter cellular metabolism and ROS production, thereby supporting tumorigenesis. Targeting these methylation pathways *in vivo* has been shown to reduce tumor burden, showing their therapeutic potential, despite limited evidence regarding m6A erasers in mitochondria.

## Conclusion

In conclusion, epigenetic and mitoepigenetic mechanisms together represent an evolving Frontier in cancer research, redefining our understanding of how gene regulation, chromatin dynamics, and mitochondrial metabolism converge to drive oncogenesis and therapeutic response. Epigenetic alterations such as DNA methylation, histone modifications, and noncoding RNAs act as reversible molecular switches that determine transcriptional plasticity, while mitoepigenetic modifications extend this regulatory logic to the mitochondrial genome, influencing oxidative phosphorylation, redox balance, apoptosis, and metabolic reprogramming. The therapeutic translation of these insights has yielded a new generation of drugs targeting epigenetic writers, readers, and erasers, from DNMT and HDAC inhibitors to EZH2 and IDH-targeted compounds, which have already improved outcomes in several hematologic and solid malignancies. However, the clinical translation of mitoepigenetic targeting remains at an early stage. Nevertheless, significant challenges remain elusive, especially the partial effectiveness of these agents in solid tumors, the development of adaptive resistance, and the limited knowledge about mitochondrial-specific epigenetic regulation in cancer progression. Over the next 5–10 years, progress in this field is likely to depend on the identification of cancer types with clear epigenetic–metabolic dependencies, the development of mitochondrial-targeted delivery systems, and the application of emerging technologies such as CRISPR/dCas9-based epigenetic editing and high-resolution multi-omics profiling. Integrating these approaches with advanced 3D tumor models and robust biomarker strategies will be essential to define context-specific vulnerabilities and to establish reproducible preclinical benchmarks. Rather than constituting an immediate paradigm shift, the convergence of epigenetic and mitoepigenetic therapies should be viewed as a progressive and evidence-driven evolution toward more personalized oncology. In this framework, therapeutic design may ultimately be guided not only by genomic alterations but also by dynamic regulatory and metabolic states that shape cancer cell identity, adaptability, and treatment response.

## Glossary

2D: two-dimensional
2-HG: 2-hydroxyglutarate
3D: three-dimensional
ADC: antibody–drug conjugate
ALL: acute lymphoblastic leukemia
ALKBH1: alkB homolog 1
AML: acute myeloid leukemia
APE1: apurinic/apyrimidinic endonuclease 1
ATAD3: ATPase family AAA-domain-containing protein 3
ATP: adenosine triphosphate
BCMA: B cell maturation antigen
BET: bromodomain and extra-terminal motif
BRD2: bromodomain-containing protein 2
BRD3: bromodomain-containing protein 3
BRD4: bromodomain-containing protein 4
BRDT: testis-specific bromodomain-containing protein
CAP: capping group (pharmacophore region of HDAC inhibitors)
CAR-T: chimeric antigen receptor T cell
CD19: cluster of differentiation 19
CD20: cluster of differentiation 20
CLDN18.2: claudin 18.2
CLL: chronic lymphocytic leukemia
CR: complete remission
CRC: colorectal cancer
CSCC: cutaneous squamous cell carcinoma
CTCL: cutaneous T cell lymphoma
dMMR: mismatch repair deficient
DNMT: DNA methyltransferase
DNMT1: DNA methyltransferase 1
DOT1L: disruptor of telomeric silencing 1-like histone methyltransferase
DSF: disulfiram
EGCG: epigallocatechin-3-gallate
EGFR: epidermal growth factor receptor
EED: embryonic ectoderm development protein
EZH1: enhancer of zeste homolog 1
EZH2: enhancer of zeste homolog 2
FDA: Food and Drug Administration
FGFR2: fibroblast growth factor receptor 2
FTO: fat mass and obesity-associated protein
GEJ: gastroesophageal junction
GIST: gastrointestinal stromal tumor
H3K4me1/2: mono- or dimethylation of lysine 4 on histone H3
H3K9me1/2: mono- or dimethylation of lysine 9 on histone H3
HAT: histone acetyltransferase
HCC: hepatocellular carcinoma
HDAC: histone deacetylase
HDACi: histone deacetylase inhibitör
HER2: human epidermal growth factor receptor 2
HMT: histone methyltransferase
HNSCC: head and neck squamous cell carcinoma
HR+: hormone receptor–positive
ICI: immune checkpoint inhibitör
IDH: isocitrate dehydrogenase
IDH1: isocitrate dehydrogenase 1
IDH2: isocitrate dehydrogenase 2
JQ1: thieno-triazolo-1,4-diazepine BET inhibitor JQ1
LIG3: DNA ligase III
LSD1: lysine-specific histone demethylase 1
MDS: myelodysplastic syndromes
METTL3: methyltransferase-like 3
miRNA: microRNA
MLL: mixed-lineage leukemia
MLL-r: mixed-lineage leukemia–rearranged
MnSOD: manganese superoxide dismutase
mtDNA: mitochondrial DNA
mtDNMT1: mitochondrial DNA methyltransferase 1
mtRNA: mitochondrial RNA
mtSSB: mitochondrial single-stranded DNA-binding protein
MUTYH: mutY DNA glycosylase homolog
m5C: 5-methylcytosine (RNA modification)
m6A: N6-methyladenosine
NAD+: nicotinamide adenine dinucleotide
NEIL1: endonuclease VIII-like 1
NEIL2: endonuclease VIII-like 2
NHL: non-Hodgkin lymphoma
NSCLC: non-small cell lung cancer
NSUN2: NOP2/Sun RNA methyltransferase 2
NSUN3: NOP2/Sun RNA methyltransferase 3
NSUN4: NOP2/Sun RNA methyltransferase 4
OGG1: 8-oxoguanine DNA glycosylase
ORR: overall response rate
OXPHOS: oxidative phosphorylation
PD-1: programmed cell death protein 1
PD-L1: programmed death ligand 1
PDGFR: platelet-derived growth factor receptor
PGC-1α: peroxisome proliferator-activated receptor gamma coactivator 1-alpha
POLG: DNA polymerase gamma
PTCL: peripheral T cell lymphoma
RCC: renal cell carcinoma
RNA: ribonucleic acid
ROS: reactive oxygen species
SCLC: small cell lung cancer
SIRT3: sirtuin 3
SIRT4: sirtuin 4
SIRT5: sirtuin 5
TET: Ten-Eleven Translocation enzyme family
TET1: Ten-Eleven Translocation methylcytosine dioxygenase 1
TET2: Ten-Eleven Translocation methylcytosine dioxygenase 2
TET3: Ten-Eleven Translocation methylcytosine dioxygenase 3
TKI: tyrosine kinase inhibitör
TNBC: triple-negative breast cancer
VEGF: vascular endothelial growth factor
WRAP53: WD repeat-containing antisense to TP53

## References

[B1] Abou-Alfa G. K. Macarulla T. Javle M. M. Kelley R. K. Lubner S. J. Adeva J. (2020). Ivosidenib in IDH1-mutant, chemotherapy-refractory cholangiocarcinoma (ClarIDHy): a multicentre, randomised, double-blind, placebo-controlled, phase 3 study. Lancet Oncol. 21, 796–807. 10.1016/s1470-2045(20)30157-1 32416072 PMC7523268

[B2] Abu Shelbayeh O. Arroum T. Morris S. Busch K. B. (2023). PGC-1α is a master regulator of mitochondrial lifecycle and ROS stress response. Antioxidants (Basel). 12, 1075. 10.3390/antiox12051075 37237941 PMC10215733

[B3] Antonia S. Goldberg S. B. Balmanoukian A. Chaft J. E. Sanborn R. E. Gupta A. (2016). Safety and antitumour activity of durvalumab plus tremelimumab in non-small cell lung cancer: a multicentre, phase 1b study. Lancet Oncol. 17, 299–308. 10.1016/s1470-2045(15)00544-6 26858122 PMC5500167

[B4] Balar A. V. Galsky M. D. Rosenberg J. E. Powles T. Petrylak D. P. Bellmunt J. (2017). Atezolizumab as first-line treatment in cisplatin-ineligible patients with locally advanced and metastatic urothelial carcinoma: a single-arm, multicentre, phase 2 trial. Lancet. 389, 67–76. 10.1016/s0140-6736(16)32455-2 27939400 PMC5568632

[B5] Bar-Yaacov D. Frumkin I. Yashiro Y. Chujo T. Ishigami Y. Chemla Y. (2016). Mitochondrial 16S rRNA is methylated by tRNA methyltransferase TRMT61B in all vertebrates. PLoS Biol. 14, e1002557. 10.1371/journal.pbio.1002557 27631568 PMC5025228

[B6] Barbato M. I. Bradford D. Ren Y. Aungst S. L. Miller C. P. Pan L. (2024). FDA approval summary: Repotrectinib for locally advanced or metastatic ROS1-Positive non-small cell lung cancer. Clin. Cancer Res. 30, 3364–3370. 10.1158/1078-0432.Ccr-24-0949 38875108 PMC11326972

[B7] Bardia A. Mayer I. A. Diamond J. R. Moroose R. L. Isakoff S. J. Starodub A. N. (2017). Efficacy and safety of Anti-Trop-2 antibody drug conjugate sacituzumab govitecan (IMMU-132) in heavily pretreated patients with metastatic triple-negative breast cancer. J. Clin. Oncol. 35, 2141–2148. 10.1200/jco.2016.70.8297 28291390 PMC5559902

[B8] Bellmunt J. Hussain M. Gschwend J. E. Albers P. Oudard S. Castellano D. (2021). Adjuvant atezolizumab versus observation in muscle-invasive urothelial carcinoma (IMvigor010): a multicentre, open-label, randomised, phase 3 trial. Lancet Oncol. 22, 525–537. 10.1016/s1470-2045(21)00004-8 33721560 PMC8495594

[B9] Berdeja J. G. Madduri D. Usmani S. Z. Jakubowiak A. Agha M. Cohen A. D. (2021). Ciltacabtagene autoleucel, a B-cell maturation antigen-directed chimeric antigen receptor T-cell therapy in patients with relapsed or refractory multiple myeloma (CARTITUDE-1): a phase 1b/2 open-label study. Lancet. 398, 314–324. 10.1016/s0140-6736(21)00933-8 34175021

[B10] Blay J. Y. Serrano C. Heinrich M. C. Zalcberg J. Bauer S. Gelderblom H. (2020). Ripretinib in patients with advanced gastrointestinal stromal tumours (INVICTUS): a double-blind, randomised, placebo-controlled, phase 3 trial. Lancet Oncol. 21, 923–934. 10.1016/s1470-2045(20)30168-6 32511981 PMC8383051

[B11] Bonner J. A. Harari P. M. Giralt J. Azarnia N. Shin D. M. Cohen R. B. (2006). Radiotherapy plus cetuximab for squamous-cell carcinoma of the head and neck. N. Engl. J. Med. 354, 567–578. 10.1056/NEJMoa053422 16467544

[B12] Breiling A. Lyko F. (2015). Epigenetic regulatory functions of DNA modifications: 5-Methylcytosine and beyond. Epigenetics Chromatin. 8, 24. 10.1186/s13072-015-0016-6 26195987 PMC4507326

[B13] Burgos Fuster L. M. Sandler A. B. (2004). Select clinical trials of erlotinib (OSI-774) in Non–small-cell lung cancer with emphasis on phase III outcomes. Clin. Lung Cancer. 6, S24–S29. 10.3816/CLC.2004.s.011 15638954

[B14] Camidge D. R. Kim H. R. Ahn M.-J. Yang J.C.-H. Han J.-Y. Lee J.-S. (2018). Brigatinib versus crizotinib in ALK-positive Non–small-cell lung cancer. N. Engl. J. Med. 379, 2027–2039. 10.1056/NEJMoa1810171 30280657

[B15] Cao K. Feng Z. Gao F. Zang W. Liu J. (2021). Mitoepigenetics: an intriguing regulatory layer in aging and metabolic-related diseases. Free Radic. Biol. Med. 177, 337–346. 10.1016/j.freeradbiomed.2021.10.031 34715295

[B16] Casali P. G. Garrett C. R. Blackstein M. E. Shah M. Verweij J. McArthur G. (2006). Updated results from a phase III trial of sunitinib in GIST patients (pts) for whom imatinib (IM) therapy has failed due to resistance or intolerance. J. Clin. Oncol. 24, 9513. 10.1200/jco.2006.24.18_suppl.9513

[B17] Celik Uzuner S. (2020). Mitochondrial DNA methylation misleads global DNA methylation detected by antibody-based methods. Anal. Biochem. 601, 113789. 10.1016/j.ab.2020.113789 32473121

[B18] Choueiri T. K. Larkin J. Oya M. Thistlethwaite F. Martignoni M. Nathan P. (2018). Preliminary results for avelumab plus axitinib as first-line therapy in patients with advanced clear-cell renal-cell carcinoma (JAVELIN renal 100): an open-label, dose-finding and dose-expansion, phase 1b trial. Lancet Oncol. 19, 451–460. 10.1016/s1470-2045(18)30107-4 29530667

[B19] Cohen M. H. Williams G. Johnson J. R. Duan J. Gobburu J. Rahman A. (2002). Approval summary for imatinib mesylate capsules in the treatment of chronic myelogenous leukemia. Clin. Cancer Res. 8, 935–942. 12006504

[B20] Connolly R. M. Zhao F. Miller K. D. Lee M. J. Piekarz R. L. Smith K. L. (2021). E2112: randomized phase III trial of endocrine therapy plus entinostat or placebo in hormone receptor-positive advanced breast cancer. A trial of the ECOG-ACRIN cancer research group. J. Clin. Oncol. 39, 3171–3181. 10.1200/jco.21.00944 34357781 PMC8478386

[B21] Coppedè F. Stoccoro A. (2019). Mitoepigenetics and neurodegenerative diseases. Front. Endocrinol. (Lausanne). 10, 86. 10.3389/fendo.2019.00086 30837953 PMC6389613

[B22] Cramer-Morales K. L. Heer C. D. Mapuskar K. A. Domann F. E. (2020). Succinate accumulation links mitochondrial MnSOD depletion to aberrant nuclear DNA methylation and altered cell fate. J. Exp. Pathol. (Wilmington). 1, 60–70. 33585836 PMC7876477

[B23] Czarna M. Jarmuszkiewicz W. (2006). Role of mitochondria in reactive oxygen species generation and removal; relevance to signaling and programmed cell death. Postepy Biochem. 52, 145–156. 17078504

[B24] Dai W. Qiao X. Fang Y. Guo R. Bai P. Liu S. (2024). Epigenetics-targeted drugs: current paradigms and future challenges. Signal Transduct. Target. Ther. 9, 332. 10.1038/s41392-024-02039-0 39592582 PMC11627502

[B25] Dey A. Chitsaz F. Abbasi A. Misteli T. Ozato K. (2003). The double bromodomain protein Brd4 binds to acetylated chromatin during interphase and mitosis. Proc. Natl. Acad. Sci. U. S. A. 100, 8758–8763. 10.1073/pnas.1433065100 12840145 PMC166386

[B26] Dhillon S. (2024). Repotrectinib: first approval. Drugs. 84, 239–246. 10.1007/s40265-023-01990-6 38279972

[B27] Dillman R. O. (1999). Perceptions of herceptin: a monoclonal antibody for the treatment of breast cancer. Cancer Biother. Radiopharm. 14, 5–10. 10.1089/cbr.1999.14.5 10850281

[B28] Dinardo M. M. Musicco C. Fracasso F. Milella F. Gadaleta M. N. Gadaleta G. (2003). Acetylation and level of mitochondrial transcription factor A in several organs of young and old rats. Biochem. Biophys. Res. Commun. 301, 187–191. 10.1016/s0006-291x(02)03008-5 12535660

[B29] DiNardo C. D. Stein E. M. de Botton S. Roboz G. J. Altman J. K. Mims A. S. (2018). Durable remissions with ivosidenib in IDH1-Mutated relapsed or refractory AML. N. Engl. J. Med. 378, 2386–2398. 10.1056/NEJMoa1716984 29860938

[B30] Dong Z. Pu L. Cui H. (2020). Mitoepigenetics and its emerging roles in cancer. Front. Cell Dev. Biol. 8, 4. 10.3389/fcell.2020.00004 32039210 PMC6989428

[B31] Du J. Zhou Y. Su X. Yu J. J. Khan S. Jiang H. (2011). Sirt5 is a NAD-dependent protein lysine demalonylase and desuccinylase. Science. 334, 806–809. 10.1126/science.1207861 22076378 PMC3217313

[B32] Duvic M. Talpur R. Ni X. Zhang C. Hazarika P. Kelly C. (2007). Phase 2 trial of oral vorinostat (suberoylanilide hydroxamic acid, SAHA) for refractory cutaneous T-cell lymphoma (CTCL). Blood. 109, 31–39. 10.1182/blood-2006-06-025999 16960145 PMC1785068

[B33] Eckersley-Maslin M. A. Alda-Catalinas C. Reik W. (2018). Dynamics of the epigenetic landscape during the maternal-to-zygotic transition. Nat. Rev. Mol. Cell Biol. 19, 436–450. 10.1038/s41580-018-0008-z 29686419

[B34] Fehrenbacher L. Spira A. Ballinger M. Kowanetz M. Vansteenkiste J. Mazieres J. (2016). Atezolizumab versus docetaxel for patients with previously treated non-small-cell lung cancer (POPLAR): a multicentre, open-label, phase 2 randomised controlled trial. Lancet. 387, 1837–1846. 10.1016/s0140-6736(16)00587-0 26970723

[B35] Fenaux P. Mufti G. J. Hellstrom-Lindberg E. Santini V. Finelli C. Giagounidis A. (2009). Efficacy of azacitidine compared with that of conventional care regimens in the treatment of higher-risk myelodysplastic syndromes: a randomised, open-label, phase III study. Lancet Oncol. 10, 223–232. 10.1016/s1470-2045(09)70003-8 19230772 PMC4086808

[B36] Fijardo M. Kwan J. Y. Y. Bissey P. A. Citrin D. E. Yip K. W. Liu F. F. (2024). The clinical manifestations and molecular pathogenesis of radiation fibrosis. EBioMedicine. 103, 105089. 10.1016/j.ebiom.2024.105089 38579363 PMC11002813

[B37] Galsky M. D. Arija Já A. Bamias A. Davis I. D. De Santis M. Kikuchi E. (2020). Atezolizumab with or without chemotherapy in metastatic urothelial cancer (IMvigor130): a multicentre, randomised, placebo-controlled phase 3 trial. Lancet. 395, 1547–1557. 10.1016/s0140-6736(20)30230-0 32416780

[B38] Ganesh K. Massagué J. (2021). Targeting metastatic cancer. Nat. Med. 27, 34–44. 10.1038/s41591-020-01195-4 33442008 PMC7895475

[B39] Garcia Campelo M. R. Zhou C. Ramalingam S. S. Lin H. M. Kim T. M. Riely G. J. (2022). Mobocertinib (TAK-788) in EGFR exon 20 insertion+ metastatic NSCLC: patient-reported outcomes from EXCLAIM extension cohort. J. Clin. Med. 12, 112. 10.3390/jcm12010112 36614913 PMC9821270

[B40] Garcia-Manero G. Kazmierczak M. Wierzbowska A. Fong C. Y. Keng M. K. Ballinari G. (2024). Pracinostat combined with azacitidine in newly diagnosed adult acute myeloid leukemia (AML) patients unfit for standard induction chemotherapy: PRIMULA phase III study. Leuk. Res. 140, 107480. 10.1016/j.leukres.2024.107480 38499457

[B41] Gilbert M. R. Dignam J. J. Armstrong T. S. Wefel J. S. Blumenthal D. T. Vogelbaum M. A. (2014). A randomized trial of bevacizumab for newly diagnosed glioblastoma. N. Engl. J. Med. 370, 699–708. 10.1056/NEJMoa1308573 24552317 PMC4201043

[B42] Gogishvili M. Melkadze T. Makharadze T. Giorgadze D. Dvorkin M. Penkov K. (2022). Cemiplimab plus chemotherapy versus chemotherapy alone in non-small cell lung cancer: a randomized, controlled, double-blind phase 3 trial. Nat. Med. 28, 2374–2380. 10.1038/s41591-022-01977-y 36008722 PMC9671806

[B43] Goldberg S. B. Gettinger S. N. Mahajan A. Chiang A. C. Herbst R. S. Sznol M. (2016). Pembrolizumab for patients with melanoma or non-small-cell lung cancer and untreated brain metastases: early analysis of a non-randomised, open-label, phase 2 trial. Lancet Oncol. 17, 976–983. 10.1016/s1470-2045(16)30053-5 27267608 PMC5526047

[B44] Gorsuch S. Bavetsias V. Rowlands M. G. Aherne G. W. Workman P. Jarman M. (2009). Synthesis of isothiazol-3-one derivatives as inhibitors of histone acetyltransferases (HATs). Bioorg Med. Chem. 17, 467–474. 10.1016/j.bmc.2008.11.079 19101154

[B45] Goyal L. Meric-Bernstam F. Hollebecque A. Valle J. W. Morizane C. Karasic T. B. (2023). Futibatinib for FGFR2-Rearranged intrahepatic cholangiocarcinoma. N. Engl. J. Med. 388, 228–239. 10.1056/NEJMoa2206834 36652354

[B46] Greene K. S. Lukey M. J. Wang X. Blank B. Druso J. E. Lin M.-c.J. (2019). SIRT5 stabilizes mitochondrial glutaminase and supports breast cancer tumorigenesis. Proc. Natl. Acad. Sci. 116, 26625–26632. 10.1073/pnas.1911954116 31843902 PMC6936584

[B47] Grillo-López A. J. White C. A. Dallaire B. K. Varns C. L. Shen C. D. Wei A. (2000). Rituximab: the first monoclonal antibody approved for the treatment of lymphoma. Curr. Pharm. Biotechnol. 1, 1–9. 10.2174/1389201003379059 11467356

[B48] Harding J. J. Fan J. Oh D. Y. Choi H. J. Kim J. W. Chang H. M. (2023). Zanidatamab for HER2-amplified, unresectable, locally advanced or metastatic biliary tract cancer (HERIZON-BTC-01): a multicentre, single-arm, phase 2b study. Lancet Oncol. 24, 772–782. 10.1016/s1470-2045(23)00242-5 37276871

[B49] Hardwick J. S. Lane A. N. Brown T. (2018). Epigenetic modifications of cytosine: biophysical properties, regulation, and function in mammalian DNA. BioEssays. 40, 1700199. 10.1002/bies.201700199 29369386

[B50] Herbst R. S. Baas P. Kim D.-W. Felip E. Pérez-Gracia J. L. Han J.-Y. (2016). Pembrolizumab versus docetaxel for previously treated, PD-L1-positive, advanced non-small-cell lung cancer (KEYNOTE-010): a randomised controlled trial. Lancet. 387, 1540–1550. 10.1016/S0140-6736(15)01281-7 26712084

[B51] Herrera A. F. LeBlanc M. Castellino S. M. Li H. Rutherford S. C. Evens A. M. (2024). Nivolumab+AVD in advanced-stage classic hodgkin's lymphoma. N. Engl. J. Med. 391, 1379–1389. 10.1056/NEJMoa2405888 39413375 PMC11488644

[B52] Hu C. Mi W. Li F. Zhu L. Ou Q. Li M. (2024). Optimizing drug combination and mechanism analysis based on risk pathway crosstalk in pan cancer. Sci. Data. 11, 74. 10.1038/s41597-024-02915-y 38228620 PMC10791624

[B53] Huang S. Hölzel M. Knijnenburg T. Schlicker A. Roepman P. McDermott U. (2012). MED12 controls the response to multiple cancer drugs through regulation of TGF-β receptor signaling. Cell. 151, 937–950. 10.1016/j.cell.2012.10.035 23178117 PMC3672971

[B54] Huang M. Huang J. Zheng Y. Sun Q. (2019). Histone acetyltransferase inhibitors: an overview in synthesis, structure-activity relationship and molecular mechanism. Eur. J. Med. Chem. 178, 259–286. 10.1016/j.ejmech.2019.05.078 31195169

[B55] Hung S. K. Lee M. S. Chiou W. Y. Liu D. W. Yu C. C. Chen L. C. (2024). Epigenetic modification in radiotherapy and immunotherapy for cancers. Tzu Chi Med. J. 36, 396–406. 10.4103/tcmj.tcmj_3_24 39421493 PMC11483092

[B56] Issa J.-P. J. Garcia-Manero G. Giles F. J. Mannari R. Thomas D. Faderl S. (2004). Phase 1 study of low-dose prolonged exposure schedules of the hypomethylating agent 5-aza-2′-deoxycytidine (decitabine) in hematopoietic malignancies. Blood. 103, 1635–1640. 10.1182/blood-2003-03-0687 14604977

[B57] Jeong S. M. Xiao C. Finley L. W. S. Lahusen T. Souza A. L. Pierce K. (2013). SIRT4 has tumor-suppressive activity and regulates the cellular metabolic response to DNA damage by inhibiting mitochondrial glutamine metabolism. Cancer Cell. 23, 450–463. 10.1016/j.ccr.2013.02.024 23562301 PMC3650305

[B58] Johnson D. H. Fehrenbacher L. Novotny W. F. Herbst R. S. Nemunaitis J. J. Jablons D. M. (2004). Randomized phase II trial comparing bevacizumab plus carboplatin and paclitaxel with carboplatin and paclitaxel alone in previously untreated locally advanced or metastatic non-small-cell lung cancer. J. Clin. Oncol. 22, 2184–2191. 10.1200/jco.2004.11.022 15169807

[B59] Johnson J. R. Cohen M. Sridhara R. Chen Y. F. Williams G. M. Duan J. (2005). Approval summary for erlotinib for treatment of patients with locally advanced or metastatic non-small cell lung cancer after failure of at least one prior chemotherapy regimen. Clin. Cancer Res. 11, 6414–6421. 10.1158/1078-0432.Ccr-05-0790 16166415

[B60] Julia E. Salles G. (2021). EZH2 inhibition by tazemetostat: mechanisms of action, safety and efficacy in relapsed/refractory follicular lymphoma. Future Oncol. 17, 2127–2140. 10.2217/fon-2020-1244 33709777 PMC9892962

[B61] Kabbinavar F. Hurwitz H. I. Fehrenbacher L. Meropol N. J. Novotny W. F. Lieberman G. (2003). Phase II, randomized trial comparing bevacizumab plus fluorouracil (FU)/leucovorin (LV) with FU/LV alone in patients with metastatic colorectal cancer. J. Clin. Oncol. 21, 60–65. 10.1200/jco.2003.10.066 12506171

[B62] Kabziński J. Walczak A. Mik M. Majsterek I. (2019). Sirt3 regulates the level of mitochondrial DNA repair activity through deacetylation of NEIL1, NEIL2, OGG1, MUTYH, APE1 and LIG3 in colorectal cancer. Pol. Przegl Chir. 92, 1–4. 10.5604/01.3001.0013.5539 32312920

[B63] Kagan A. B. Garrison D. A. Anders N. M. Webster J. A. Baker S. D. Yegnasubramanian S. (2023). DNA methyltransferase inhibitor exposure-response: challenges and opportunities. Clin. Transl. Sci. 16, 1309–1322. 10.1111/cts.13548 37345219 PMC10432879

[B64] Kantarjian H. Issa J. P. Rosenfeld C. S. Bennett J. M. Albitar M. DiPersio J. (2006). Decitabine improves patient outcomes in myelodysplastic syndromes: results of a phase III randomized study. Cancer. 106, 1794–1803. 10.1002/cncr.21792 16532500

[B65] Kaplánek R. Kejík Z. Hajduch J. Veselá K. Kučnirová K. Skaličková M. (2023). TET protein inhibitors: potential and limitations. Biomed. Pharmacother. 166, 115324. 10.1016/j.biopha.2023.115324 37598475

[B66] Katanić Stanković J. S. Selaković D. Rosić G. (2023). Oxidative damage as a fundament of systemic toxicities induced by cisplatin—the crucial limitation or potential therapeutic target? Int. J. Mol. Sci. 24, 14574. 10.3390/ijms241914574 37834021 PMC10572959

[B67] Kaufman H. L. Russell J. Hamid O. Bhatia S. Terheyden P. D'Angelo S. P. (2016). Avelumab in patients with chemotherapy-refractory metastatic merkel cell carcinoma: a multicentre, single-group, open-label, phase 2 trial. Lancet Oncol. 17, 1374–1385. 10.1016/s1470-2045(16)30364-3 27592805 PMC5587154

[B68] Kelley R. K. Sangro B. Harris W. Ikeda M. Okusaka T. Kang Y. K. (2021). Efficacy, and pharmacodynamics of tremelimumab plus durvalumab for patients with unresectable hepatocellular carcinoma: randomized expansion of a phase I/II study. J. Clin. Oncol. 39, 2991–3001. 10.1200/jco.20.03555 34292792 PMC8445563

[B69] Khozin S. Weinstock C. Blumenthal G. M. Cheng J. He K. Zhuang L. (2017). Osimertinib for the treatment of metastatic EGFR T790M mutation-positive non-small cell lung cancer. Clin. Cancer Res. 23, 2131–2135. 10.1158/1078-0432.Ccr-16-1773 27923840

[B70] King G. A. Hashemi Shabestari M. Taris K. H. Pandey A. K. Venkatesh S. Thilagavathi J. (2018). Acetylation and phosphorylation of human TFAM regulate TFAM-DNA interactions via contrasting mechanisms. Nucleic Acids Res. 46, 3633–3642. 10.1093/nar/gky204 29897602 PMC5909435

[B71] Koka K. Verma A. Dwarakanath B. S. Papineni R. V. L. (2022). Technological advancements in external beam radiation therapy (EBRT): an indispensable tool for cancer treatment. Cancer Manag. Res. 14, 1421–1429. 10.2147/cmar.S351744 35431581 PMC9012312

[B72] Kopetz S. Yoshino T. Van Cutsem E. Eng C. Kim T. W. Wasan H. S. (2025). Encorafenib, cetuximab and chemotherapy in BRAF-mutant colorectal cancer: a randomized phase 3 trial. Nat. Med. 31, 901–908. 10.1038/s41591-024-03443-3 39863775 PMC11922750

[B73] Krop I. E. LoRusso P. Miller K. D. Modi S. Yardley D. Rodriguez G. (2012). A phase II study of trastuzumab emtansine in patients with human epidermal growth factor receptor 2-positive metastatic breast cancer who were previously treated with trastuzumab, lapatinib, an anthracycline, a taxane, and capecitabine. J. Clin. Oncol. 30, 3234–3241. 10.1200/jco.2011.40.5902 22649126

[B74] Lee H. Z. Kwitkowski V. E. Del Valle P. L. Ricci M. S. Saber H. Habtemariam B. A. (2015). FDA approval: Belinostat for the treatment of patients with relapsed or refractory peripheral T-cell lymphoma. Clin. Cancer Res. 21, 2666–2670. 10.1158/1078-0432.Ccr-14-3119 25802282

[B75] Lei T. Rui Y. Xiaoshuang Z. Jinglan Z. Jihong Z. (2024). Mitochondria transcription and cancer. Cell Death Discov. 10, 168. 10.1038/s41420-024-01926-3 38589371 PMC11001877

[B76] Lembo V. Bottegoni G. (2024). Systematic investigation of dual-target-directed ligands. J. Med. Chem. 67, 10374–10385. 10.1021/acs.jmedchem.4c00838 38843874 PMC11215722

[B77] Lenz H. J. Van Cutsem E. Luisa Limon M. Wong K. Y. M. Hendlisz A. Aglietta M. (2022). First-line nivolumab plus low-dose ipilimumab for microsatellite instability-High/Mismatch repair-deficient metastatic colorectal cancer: the phase II CheckMate 142 study. J. Clin. Oncol. 40, 161–170. 10.1200/jco.21.01015 34637336

[B78] Li M. Tao Z. Zhao Y. Li L. Zheng J. Li Z. (2022). 5-methylcytosine RNA methyltransferases and their potential roles in cancer. J. Transl. Med. 20, 214. 10.1186/s12967-022-03427-2 35562754 PMC9102922

[B79] Li J. Gong C. Zhou H. Liu J. Xia X. Ha W. (2024). Kinase inhibitors and kinase-targeted cancer therapies: recent advances and future perspectives. Int. J. Mol. Sci. 25, 5489. 10.3390/ijms25105489 38791529 PMC11122109

[B80] Li F. Feng Y. Yin Z. Wang Y. (2025). Mitochondrial metabolism in T-Cell exhaustion. Int. J. Mol. Sci. 26, 7400. 10.3390/ijms26157400 40806529 PMC12347488

[B81] Liu Y. Yang Q. (2023). The roles of EZH2 in cancer and its inhibitors. Med. Oncol. 40, 167. 10.1007/s12032-023-02025-6 37148376 PMC10162908

[B82] Liu Y. P. Zheng C. C. Huang Y. N. He M. L. Xu W. W. Li B. (2021). Molecular mechanisms of chemo- and radiotherapy resistance and the potential implications for cancer treatment. MedComm (2020). 2 (2), 315–340. 10.1002/mco2.55 34766149 PMC8554658

[B83] Liu D. Wang Y. Jing H. Meng Q. Yang J. (2022). Novel DNA methylation loci and genes showing pleiotropic association with Alzheimer's dementia: a network Mendelian randomization analysis. Epigenetics. 17, 746–758. 10.1080/15592294.2021.1959735 34461811 PMC9336475

[B84] Liu B. Zhou H. Tan L. Siu K. T. H. Guan X.-Y. (2024a). Exploring treatment options in cancer: tumor treatment strategies. Signal Transduct. Target. Ther. 9, 175. 10.1038/s41392-024-01856-7 39013849 PMC11252281

[B85] Liu Z. Chen J. Ren Y. Liu S. Ba Y. Zuo A. (2024b). Multi-stage mechanisms of tumor metastasis and therapeutic strategies. Signal Transduct. Target. Ther. 9, 270. 10.1038/s41392-024-01955-5 39389953 PMC11467208

[B86] Lordick F. Van Cutsem E. Shitara K. Xu R. H. Ajani J. A. Shah M. A. (2024). Health-related quality of life in patients with CLDN18.2-positive, locally advanced unresectable or metastatic gastric or gastroesophageal junction adenocarcinoma: results from the SPOTLIGHT and GLOW clinical trials. ESMO Open. 9, 103663. 10.1016/j.esmoop.2024.103663 39146670 PMC11374961

[B87] Lu X. Ning Z. Li Z. Cao H. Wang X. (2016). Development of chidamide for peripheral T-cell lymphoma, the first orphan drug approved in China. Intractable Rare Dis. Res. 5, 185–191. 10.5582/irdr.2016.01024 27672541 PMC4995415

[B88] Lu S. Kato T. Dong X. Ahn M.-J. Quang L.-V. Soparattanapaisarn N. (2024). Osimertinib after chemoradiotherapy in stage III *EGFR*-mutated NSCLC. N. Engl. J. Med. 391, 585–597. 10.1056/NEJMoa2402614 38828946

[B89] Makita S. Yamamoto G. Maruyama D. Asano-Mori Y. Kaji D. Ananthakrishnan R. (2022). Phase 2 results of lisocabtagene maraleucel in Japanese patients with relapsed/refractory aggressive B-cell non-Hodgkin lymphoma. Cancer Med. 11, 4889–4899. 10.1002/cam4.4820 35619325 PMC9761090

[B90] Marabelle A. Le D. T. Ascierto P. A. Di Giacomo A. M. De Jesus-Acosta A. Delord J. P. (2020). Efficacy of pembrolizumab in patients with noncolorectal high microsatellite instability/mismatch repair-deficient cancer: results from the phase II KEYNOTE-158 study. J. Clin. Oncol. 38, 1–10. 10.1200/jco.19.02105 31682550 PMC8184060

[B91] Martínez-Reyes I. Chandel N. S. (2020). Mitochondrial TCA cycle metabolites control physiology and disease. Nat. Commun. 11, 102. 10.1038/s41467-019-13668-3 31900386 PMC6941980

[B92] Mellinghoff I. K. van den Bent M. J. Blumenthal D. T. Touat M. Peters K. B. Clarke J. (2023a). Vorasidenib in IDH1- or IDH2-Mutant low-grade glioma. N. Engl. J. Med. 389, 589–601. 10.1056/NEJMoa2304194 37272516 PMC11445763

[B93] Mellinghoff I. K. Lu M. Wen P. Y. Taylor J. W. Maher E. A. Arrillaga-Romany I. (2023b). Vorasidenib and ivosidenib in IDH1-mutant low-grade glioma: a randomized, perioperative phase 1 trial. Nat. Med. 29, 615–622. 10.1038/s41591-022-02141-2 36823302 PMC10313524

[B94] Menghrajani K. Cai S. F. Devlin S. M. Armstrong S. A. Piekarz R. Rudek M. (2019). A phase Ib/II study of the histone methyltransferase inhibitor pinometostat in combination with azacitidine in patients with 11q23-Rearranged acute myeloid leukemia. Blood. 134, 2655. 10.1182/blood-2019-121926

[B95] Meric-Bernstam F. Beeram M. Hamilton E. Oh D. Y. Hanna D. L. Kang Y. K. (2022). Zanidatamab, a novel bispecific antibody, for the treatment of locally advanced or metastatic HER2-expressing or HER2-amplified cancers: a phase 1, dose-escalation and expansion study. Lancet Oncol. 23, 1558–1570. 10.1016/s1470-2045(22)00621-0 36400106

[B96] Migden M. R. Rischin D. Schmults C. D. Guminski A. Hauschild A. Lewis K. D. (2018). PD-1 blockade with cemiplimab in advanced cutaneous squamous-cell carcinoma. N. Engl. J. Med. 379, 341–351. 10.1056/NEJMoa1805131 29863979

[B97] Min H. Y. Lee H. Y. (2022). Molecular targeted therapy for anticancer treatment. Exp. Mol. Med. 54, 1670–1694. 10.1038/s12276-022-00864-3 36224343 PMC9636149

[B98] Mirza M. R. Chase D. M. Slomovitz B. M. dePont Christensen R. Novák Z. Black D. (2023). Dostarlimab for primary advanced or recurrent endometrial cancer. N. Engl. J. Med. 388, 2145–2158. 10.1056/NEJMoa2216334 36972026

[B99] Mittendorf E. A. Zhang H. Barrios C. H. Saji S. Jung K. H. Hegg R. (2020). Neoadjuvant atezolizumab in combination with sequential nab-paclitaxel and anthracycline-based chemotherapy versus placebo and chemotherapy in patients with early-stage triple-negative breast cancer (IMpassion031): a randomised, double-blind, phase 3 trial. Lancet. 396, 1090–1100. 10.1016/s0140-6736(20)31953-x 32966830

[B100] Moore M. J. Goldstein D. Hamm J. Figer A. Hecht J. R. Gallinger S. (2007). Erlotinib plus gemcitabine compared with gemcitabine alone in patients with advanced pancreatic cancer: a phase III trial of the national cancer institute of Canada clinical trials group. J. Clin. Oncol. 25, 1960–1966. 10.1200/jco.2006.07.9525 17452677

[B101] Mohan N. (2025). Nucleoside and non-nucleoside DNA methyltransferase 1 inhibitors in epigenetic and combination therapies in cancer: a scoping review. Undergrad. Res. Nat. Clin. Sci. Technol. (URNCST) J. 9, 1–8. 10.26685/urncst.641

[B102] Morschhauser F. Tilly H. Chaidos A. McKay P. Phillips T. Assouline S. (2020). Tazemetostat for patients with relapsed or refractory follicular lymphoma: an open-label, single-arm, multicentre, phase 2 trial. Lancet Oncol. 21, 1433–1442. 10.1016/s1470-2045(20)30441-1 33035457 PMC8427481

[B103] Mosca L. Vitiello F. Borzacchiello L. Coppola A. Tranchese R. V. Pagano M. (2021). Mutual correlation between non-coding RNA and S-Adenosylmethionine in human cancer: roles and therapeutic opportunities. Cancers (Basel). 13, 3264. 10.3390/cancers13133264 34209866 PMC8268931

[B104] Motzer R. J. Hutson T. E. Tomczak P. Michaelson M. D. Bukowski R. M. Rixe O. (2007). Sunitinib versus interferon alfa in metastatic renal-cell carcinoma. N. Engl. J. Med. 356, 115–124. 10.1056/NEJMoa065044 17215529

[B105] Motzer R. J. Escudier B. McDermott D. F. George S. Hammers H. J. Srinivas S. (2015). Nivolumab versus everolimus in advanced renal-cell carcinoma. N. Engl. J. Med. 373, 1803–1813. 10.1056/NEJMoa1510665 26406148 PMC5719487

[B106] Munshi N. C. Anderson L. D. Jr. Shah N. Madduri D. Berdeja J. Lonial S. (2021). Idecabtagene vicleucel in relapsed and refractory multiple myeloma. N. Engl. J. Med. 384, 705–716. 10.1056/NEJMoa2024850 33626253

[B107] Nagaya T. Nakamura Y. A. Choyke P. L. Kobayashi H. (2017). Fluorescence-guided surgery. Front. Oncol. 7, 314. 10.3389/fonc.2017.00314 29312886 PMC5743791

[B108] Neelapu S. S. Locke F. L. Bartlett N. L. Lekakis L. J. Miklos D. B. Jacobson C. A. (2017). Axicabtagene ciloleucel CAR T-Cell therapy in refractory large B-Cell lymphoma. N. Engl. J. Med. 377, 2531–2544. 10.1056/NEJMoa1707447 29226797 PMC5882485

[B109] Neganova M. E. Klochkov S. G. Aleksandrova Y. R. Aliev G. (2022). Histone modifications in epigenetic regulation of cancer: perspectives and achieved progress. Semin. Cancer Biol. 83, 452–471. 10.1016/j.semcancer.2020.07.015 32814115

[B110] Nitsch S. Zorro Shahidian L. Schneider R. (2021). Histone acylations and chromatin dynamics: concepts, challenges, and links to metabolism. EMBO Reports. 22, e52774. 10.15252/embr.202152774 34159701 PMC8406397

[B111] Noce B. Di Bello E. Fioravanti R. Mai A. (2023). LSD1 inhibitors for cancer treatment: focus on multi-target agents and compounds in clinical trials. Front. Pharmacol. 14, 14–2023. 10.3389/fphar.2023.1120911 36817147 PMC9932783

[B112] Nouri Z. Fakhri S. Nouri K. Wallace C. E. Farzaei M. H. Bishayee A. (2020). Targeting multiple signaling pathways in cancer: the rutin therapeutic approach. Cancers (Basel). 12. 10.3390/cancers12082276 32823876 PMC7463935

[B113] O'Leary M. C. Lu X. Huang Y. Lin X. Mahmood I. Przepiorka D. (2019). FDA approval summary: tisagenlecleucel for treatment of patients with relapsed or refractory B-cell precursor acute lymphoblastic leukemia. Clin. Cancer Res. 25, 1142–1146. 10.1158/1078-0432.Ccr-18-2035 30309857

[B114] Oh D. Y. He A. R. Bouattour M. Okusaka T. Qin S. Chen L. T. (2024). Durvalumab or placebo plus gemcitabine and cisplatin in participants with advanced biliary tract cancer (TOPAZ-1): updated overall survival from a randomised phase 3 study. Lancet Gastroenterol. Hepatol. 9, 694–704. 10.1016/s2468-1253(24)00095-5 38823398

[B115] Oliveira M. Rugo H. S. Howell S. J. Dalenc F. Cortes J. Gomez H. L. (2024). Capivasertib and fulvestrant for patients with hormone receptor-positive, HER2-negative advanced breast cancer (CAPItello-291): patient-reported outcomes from a phase 3, randomised, double-blind, placebo-controlled trial. Lancet Oncol. 25, 1231–1244. 10.1016/s1470-2045(24)00373-5 39214106

[B116] Olsen E. A. Kim Y. H. Kuzel T. M. Pacheco T. R. Foss F. M. Parker S. (2007). Phase IIb multicenter trial of vorinostat in patients with persistent, progressive, or treatment refractory cutaneous T-cell lymphoma. J. Clin. Oncol. 25, 3109–3115. 10.1200/jco.2006.10.2434 17577020

[B117] Pan J. Liu F. Xiao X. Xu R. Dai L. Zhu M. (2022). METTL3 promotes colorectal carcinoma progression by regulating the m6A–CRB3–Hippo axis. J. Exp. and Clin. Cancer Res. 41, 19. 10.1186/s13046-021-02227-8 35012593 PMC8744223

[B118] Pandey P. Verma M. Lakhanpal S. Pandey S. Kumar M. R. Bhat M. (2025). An updated review summarizing the anticancer potential of Poly(Lactic-co-Glycolic acid) (PLGA) based curcumin, epigallocatechin gallate, and resveratrol nanocarriers. Biopolymers. 116, e23637. 10.1002/bip.23637 39417679

[B119] Patel M. R. Ellerton J. Infante J. R. Agrawal M. Gordon M. Aljumaily R. (2018). Avelumab in metastatic urothelial carcinoma after platinum failure (JAVELIN solid tumor): pooled results from two expansion cohorts of an open-label, phase 1 trial. Lancet Oncol. 19, 51–64. 10.1016/s1470-2045(17)30900-2 29217288 PMC7984727

[B120] Paz-Ares L. Dvorkin M. Chen Y. Reinmuth N. Hotta K. Trukhin D. (2019). Durvalumab plus platinum-etoposide versus platinum-etoposide in first-line treatment of extensive-stage small-cell lung cancer (CASPIAN): a randomised, controlled, open-label, phase 3 trial. Lancet. 394, 1929–1939. 10.1016/s0140-6736(19)32222-6 31590988

[B121] Piekarz R. L. Frye R. Turner M. Wright J. J. Allen S. L. Kirschbaum M. H. (2009). Phase II multi-institutional trial of the histone deacetylase inhibitor romidepsin as monotherapy for patients with cutaneous T-cell lymphoma. J. Clin. Oncol. 27, 5410–5417. 10.1200/jco.2008.21.6150 19826128 PMC2773225

[B122] Piekarz R. L. Frye R. Prince H. M. Kirschbaum M. H. Zain J. Allen S. L. (2011). Phase 2 trial of romidepsin in patients with peripheral T-cell lymphoma. Blood. 117, 5827–5834. 10.1182/blood-2010-10-312603 21355097 PMC3112033

[B123] Planchard D. Yokoi T. McCleod M. J. Fischer J. R. Kim Y. C. Ballas M. (2016). A phase III study of durvalumab (MEDI4736) with or without tremelimumab for previously treated patients with advanced NSCLC: rationale and protocol design of the ARCTIC study. Clin. Lung Cancer. 17, 232–236.e231. 10.1016/j.cllc.2016.03.003 27265743

[B124] Poole R. M. (2014). Belinostat: first global approval. Drugs. 74, 1543–1554. 10.1007/s40265-014-0275-8 25134672

[B125] Pourzia A. L. Olson M. L. Bailey S. R. Boroughs A. C. Aryal A. Ryan J. (2023). Quantifying requirements for mitochondrial apoptosis in CAR T killing of cancer cells. Cell Death and Dis. 14, 267. 10.1038/s41419-023-05727-x 37055388 PMC10101951

[B126] Puppi M. Sacchetti I. Mancuso K. Tacchetti P. Pantani L. Rizzello I. (2025). Bispecific antibodies and CAR T in multiple myeloma: appropriate selection of patients and sequencing. Mediterr. J. Hematol. Infect. Dis. 17, e2025045. 10.4084/mjhid.2025.045 40375902 PMC12081044

[B127] Qin S. Chen M. Cheng A. L. Kaseb A. O. Kudo M. Lee H. C. (2023). Atezolizumab plus bevacizumab versus active surveillance in patients with resected or ablated high-risk hepatocellular carcinoma (IMbrave050): a randomised, open-label, multicentre, phase 3 trial. Lancet. 402, 1835–1847. 10.1016/s0140-6736(23)01796-8 37871608

[B128] Qiu M. Z. Oh D. Y. Kato K. Arkenau T. Tabernero J. Correa M. C. (2024). Tislelizumab plus chemotherapy versus placebo plus chemotherapy as first line treatment for advanced gastric or gastro-oesophageal junction adenocarcinoma: RATIONALE-305 randomised, double blind, phase 3 trial. Bmj. 385, e078876. 10.1136/bmj-2023-078876 38806195

[B129] Rad S. M. A. Halpin J. C. Mollaei M. Smith Bell S. W. J. Hirankarn N. McLellan A. D. (2021). Metabolic and mitochondrial functioning in chimeric antigen receptor (CAR)-T cells. Cancers (Basel). 13. 10.3390/cancers13061229 PMC8002030 33799768

[B130] Rando O. J. (2012). Combinatorial complexity in chromatin structure and function: revisiting the histone code. Curr. Opin. Genet. and Dev. 22, 148–155. 10.1016/j.gde.2012.02.013 22440480 PMC3345062

[B131] Raymond E. Dahan L. Raoul J.-L. Bang Y.-J. Borbath I. Lombard-Bohas C. (2011). Sunitinib malate for the treatment of pancreatic neuroendocrine tumors. N. Engl. J. Med. 364, 501–513. 10.1056/NEJMoa1003825 21306237

[B132] Reid M. A. Dai Z. Locasale J. W. (2017). The impact of cellular metabolism on chromatin dynamics and epigenetics. Nat. Cell Biol. 19, 1298–1306. 10.1038/ncb3629 29058720 PMC5886854

[B133] Rizvi N. A. Mazières J. Planchard D. Stinchcombe T. E. Dy G. K. Antonia S. J. (2015). Activity and safety of nivolumab, an anti-PD-1 immune checkpoint inhibitor, for patients with advanced, refractory squamous non-small-cell lung cancer (checkMate 063): a phase 2, single-arm trial. Lancet Oncol. 16, 257–265. 10.1016/s1470-2045(15)70054-9 25704439 PMC5726228

[B134] Rosenberg J. E. Hoffman-Censits J. Powles T. van der Heijden M. S. Balar A. V. Necchi A. (2016). Atezolizumab in patients with locally advanced and metastatic urothelial carcinoma who have progressed following treatment with platinum-based chemotherapy: a single-arm, multicentre, phase 2 trial. Lancet. 387, 1909–1920. 10.1016/s0140-6736(16)00561-4 26952546 PMC5480242

[B135] Royce M. Shah M. Zhang L. Cheng J. Bonner M. K. Pegues M. (2025). FDA approval summary: Datopotamab deruxtecan-dlnk for treatment of patients with unresectable or metastatic, HR-positive, HER2-negative breast cancer. Clin. Cancer Res. 31, 4405–4411. 10.1158/1078-0432.Ccr-25-1388 40864501 PMC12393668

[B136] Rugo H. S. Bardia A. Marmé F. Cortés J. Schmid P. Loirat D. (2023). Overall survival with sacituzumab govitecan in hormone receptor-positive and human epidermal growth factor receptor 2-negative metastatic breast cancer (TROPiCS-02): a randomised, open-label, multicentre, phase 3 trial. Lancet. 402, 1423–1433. 10.1016/s0140-6736(23)01245-x 37633306

[B137] Rugo H. S. Oliveira M. Howell S. J. Dalenc F. Cortes J. Gomez H. L. (2024). Capivasertib and fulvestrant for patients with hormone receptor-positive advanced breast cancer: characterization, time course, and management of frequent adverse events from the phase III CAPItello-291 study. ESMO Open. 9, 103697. 10.1016/j.esmoop.2024.103697 39241495 PMC11406080

[B138] Ryszkiewicz P. Malinowska B. Schlicker E. (2025). Polypharmacology: new drugs in 2023–2024. Pharmacol. Rep. 77, 543–560. 10.1007/s43440-025-00715-8 40095348 PMC12066383

[B139] Santos J. M. Mishra M. Kowluru R. A. (2014). Posttranslational modification of mitochondrial transcription factor A in impaired mitochondria biogenesis: implications in diabetic retinopathy and metabolic memory phenomenon. Exp. Eye Res. 121, 168–177. 10.1016/j.exer.2014.02.010 24607487 PMC4036231

[B140] Seiwert T. Y. Burtness B. Mehra R. Weiss J. Berger R. Eder J. P. (2016). Safety and clinical activity of pembrolizumab for treatment of recurrent or metastatic squamous cell carcinoma of the head and neck (KEYNOTE-012): an open-label, multicentre, phase 1b trial. Lancet Oncol. 17, 956–965. 10.1016/S1470-2045(16)30066-3 27247226

[B141] Shah V. McNatty A. Simpson L. Ofori H. Raheem F. (2023). Amivantamab-vmjw: a novel treatment for patients with NSCLC harboring EGFR exon 20 insertion mutation after progression on platinum-based chemotherapy. Biomedicines. 11, 950. 10.3390/biomedicines11030950 36979929 PMC10046583

[B142] Shang R. Lee S. Senavirathne G. Lai E. C. (2023). microRNAs in action: biogenesis, function and regulation. Nat. Rev. Genet. 24, 816–833. 10.1038/s41576-023-00611-y 37380761 PMC11087887

[B143] Sharma S. V. Lee D. Y. Li B. Quinlan M. P. Takahashi F. Maheswaran S. (2010). A chromatin-mediated reversible drug-tolerant state in cancer cell subpopulations. Cell. 141, 69–80. 10.1016/j.cell.2010.02.027 20371346 PMC2851638

[B144] Shepherd F. A. Pereira J. Ciuleanu T. E. Tan E. H. Hirsh V. Thongprasert S. (2004). A randomized placebo-controlled trial of erlotinib in patients with advanced non-small cell lung cancer (NSCLC) following failure of 1^st^ line or 2^nd^ line chemotherapy. A national cancer institute of Canada clinical trials group (NCIC CTG) trial. J. Clin. Oncol. 22, 7022. 10.1200/jco.2004.22.90140.7022

[B145] Shi Y. Jia B. Xu W. Li W. Liu T. Liu P. (2017). Chidamide in relapsed or refractory peripheral T cell lymphoma: a multicenter real-world study in China. J. Hematol. Oncol. 10, 69. 10.1186/s13045-017-0439-6 28298231 PMC5351273

[B146] Shock L. S. Thakkar P. V. Peterson E. J. Moran R. G. Taylor S. M. (2011). DNA methyltransferase 1, cytosine methylation, and cytosine hydroxymethylation in Mammalian mitochondria. Proc. Natl. Acad. Sci. U. S. A. 108, 3630–3635. 10.1073/pnas.1012311108 21321201 PMC3048134

[B147] Sirard M.-A. (2019). Distribution and dynamics of mitochondrial DNA methylation in oocytes, embryos and granulosa cells. Sci. Rep. 9, 11937. 10.1038/s41598-019-48422-8 31417147 PMC6695495

[B148] Smalley J. P. Cowley S. M. Hodgkinson J. T. (2020). Bifunctional HDAC therapeutics: one drug to rule them all? Molecules. 25. 10.3390/molecules25194394 32987782 PMC7583022

[B149] Song Y. Zhu X. Y. Zhang X. M. Xiong H. (2022). Targeted mitochondrial epigenetics: a new direction in Alzheimer's disease treatment. Int. J. Mol. Sci. 23, 9703. 10.3390/ijms23179703 36077101 PMC9456144

[B150] Soria J.-C. Ohe Y. Vansteenkiste J. Reungwetwattana T. Chewaskulyong B. Lee K. H. (2018). Osimertinib in untreated EGFR-mutated advanced Non–small-cell lung cancer. N. Engl. J. Med. 378, 113–125. 10.1056/NEJMoa1713137 29151359

[B151] Stein E. M. DiNardo C. D. Pollyea D. A. Fathi A. T. Roboz G. J. Altman J. K. (2017). Enasidenib in mutant IDH2 relapsed or refractory acute myeloid leukemia. Blood. 130, 722–731. 10.1182/blood-2017-04-779405 28588020 PMC5572791

[B152] Stein E. M. Garcia-Manero G. Rizzieri D. A. Tibes R. Berdeja J. G. Savona M. R. (2018). The DOT1L inhibitor pinometostat reduces H3K79 methylation and has modest clinical activity in adult acute leukemia. Blood. 131, 2661–2669. 10.1182/blood-2017-12-818948 29724899 PMC6265654

[B153] Stoccoro A. Coppedè F. (2021). Mitochondrial DNA methylation and human diseases. Int. J. Mol. Sci. 22, 4594. 10.3390/ijms22094594 33925624 PMC8123858

[B154] Sumiyoshi A. Shibata S. Zhelev Z. Miller T. Lazarova D. Aoki I. (2022). Targeting glioblastoma via selective alteration of mitochondrial redox state. Cancers (Basel). 14, 485. 10.3390/cancers14030485 35158753 PMC8833725

[B155] Sun X. Vaghjiani V. Jayasekara W. S. N. Cain J. E. St John J. C. (2018). The degree of mitochondrial DNA methylation in tumor models of glioblastoma and osteosarcoma. Clin. Epigenetics. 10, 157. 10.1186/s13148-018-0590-0 30558637 PMC6296150

[B156] Suraweera A. O’Byrne K. J. Richard D. J. (2025). Epigenetic drugs in cancer therapy. Cancer Metastasis Rev. 44, 37. 10.1007/s10555-025-10253-7 40011240 PMC11865116

[B157] Swain S. M. Shastry M. Hamilton E. (2023). Targeting HER2-positive breast cancer: advances and future directions. Nat. Rev. Drug Discov. 22, 101–126. 10.1038/s41573-022-00579-0 36344672 PMC9640784

[B158] To K. K. W. Xing E. Larue R. C. Li P. K. (2023). BET bromodomain inhibitors: novel design strategies and therapeutic applications. Molecules. 28, 3043. 10.3390/molecules28073043 37049806 PMC10096006

[B159] Torrens-Mas M. Hernández-López R. Pons D.-G. Roca P. Oliver J. Sastre-Serra J. (2019). Sirtuin 3 silencing impairs mitochondrial biogenesis and metabolism in colon cancer cells. Am. J. Physiology-Cell Physiology. 317, C398–C404. 10.1152/ajpcell.00112.2019 31188638

[B160] Turner N. C. Oliveira M. Howell S. J. Dalenc F. Cortes J. Gomez Moreno H. L. (2023). Capivasertib in hormone receptor-positive advanced breast cancer. N. Engl. J. Med. 388, 2058–2070. 10.1056/NEJMoa2214131 37256976 PMC11335038

[B161] Tykodi S. S. Gordan L. N. Alter R. S. Arrowsmith E. Harrison M. R. Percent I. (2022). Safety and efficacy of nivolumab plus ipilimumab in patients with advanced non-clear cell renal cell carcinoma: results from the phase 3b/4 CheckMate 920 trial. J. Immunother. Cancer 10, e003844. 10.1136/jitc-2021-003844 35210307 PMC8883262

[B162] Van Haute L. Dietmann S. Kremer L. Hussain S. Pearce S. F. Powell C. A. (2016). Deficient methylation and formylation of mt-tRNAMet wobble cytosine in a patient carrying mutations in NSUN3. Nat. Commun. 7, 12039. 10.1038/ncomms12039 27356879 PMC4931328

[B163] Verweij J. van Oosterom A. Blay J. Y. Judson I. Rodenhuis S. van der Graaf W. (2003). Imatinib mesylate (STI-571 glivec, gleevec) is an active agent for gastrointestinal stromal tumours, but does not yield responses in other soft-tissue sarcomas that are unselected for a molecular target. Results from an EORTC soft tissue and bone sarcoma group phase II study. Eur. J. Cancer. 39, 2006–2011. 10.1016/S0959-8049(02)00836-5 12957454

[B164] Wang L. Zhou Y. Xu L. Xiao R. Lu X. Chen L. (2015). Molecular basis for 5-carboxycytosine recognition by RNA polymerase II elongation complex. Nature. 523, 621–625. 10.1038/nature14482 26123024 PMC4521995

[B165] Wang J. Zhang Q. Hu X. Li Q. Sun T. Li W. (2024). a class I selective histone deacetylase inhibitor, plus exemestane for Chinese patients with hormone receptor-positive advanced breast cancer: an overall survival update and long-term safety from the randomised, double-blind, placebo-controlled, phase 3 trial. J. Clin. Oncol. 42, 1050. 10.1200/JCO.2024.42.16_suppl.1050

[B166] Wang R. Hu B. Pan Z. Mo C. Zhao X. Liu G. (2025). Antibody–drug conjugates (ADCs): current and future biopharmaceuticals. J. Hematol. and Oncol. 18, 51. 10.1186/s13045-025-01704-3 40307936 PMC12044742

[B167] Weber J. S. D'Angelo S. P. Minor D. Hodi F. S. Gutzmer R. Neyns B. (2015). Nivolumab versus chemotherapy in patients with advanced melanoma who progressed after anti-CTLA-4 treatment (checkMate 037): a randomised, controlled, open-label, phase 3 trial. Lancet Oncol. 16, 375–384. 10.1016/s1470-2045(15)70076-8 25795410

[B168] Wolchok J. D. Neyns B. Linette G. Negrier S. Lutzky J. Thomas L. (2010). Ipilimumab monotherapy in patients with pretreated advanced melanoma: a randomised, double-blind, multicentre, phase 2, dose-ranging study. Lancet Oncol. 11, 155–164. 10.1016/s1470-2045(09)70334-1 20004617

[B169] Xu J. Kato K. Raymond E. Hubner R. A. Shu Y. Pan Y. (2023). Tislelizumab plus chemotherapy versus placebo plus chemotherapy as first-line treatment for advanced or metastatic oesophageal squamous cell carcinoma (RATIONALE-306): a global, randomised, placebo-controlled, phase 3 study. Lancet Oncol. 24, 483–495. 10.1016/s1470-2045(23)00108-0 37080222

[B170] Yalniz F. F. Berdeja J. G. Maris M. B. Lyons R. M. Reeves J. A. Jr. Essell J. H. (2020). A phase II study of addition of pracinostat to a hypomethylating agent in patients with myelodysplastic syndromes who have not responded to previous hypomethylating agent therapy. Br. J. Haematol. 188, 404–412. 10.1111/bjh.16173 31468521

[B171] Yamashita T. Sohn J. H. Tokunaga E. Niikura N. Park Y. H. Lee K. S. (2024). Trastuzumab deruxtecan versus treatment of physician's choice in previously treated Asian patients with HER2-low unresectable/metastatic breast cancer: subgroup analysis of the DESTINY-Breast04 study. Breast Cancer. 31, 858–868. 10.1007/s12282-024-01600-7 38884900 PMC11341650

[B172] Yang J. C. Haworth L. Sherry R. M. Hwu P. Schwartzentruber D. J. Topalian S. L. (2003). A randomized trial of bevacizumab, an anti-vascular endothelial growth factor antibody, for metastatic renal cancer. N. Engl. J. Med. 349, 427–434. 10.1056/NEJMoa021491 12890841 PMC2275324

[B173] Yeruva S. L. H. Zhao F. Miller K. D. Tevaarwerk A. J. Wagner L. I. Gray R. J. (2018). E2112: randomized phase iii trial of endocrine therapy plus entinostat/placebo in patients with hormone receptor-positive advanced breast cancer. NPJ Breast Cancer. 4, 1. 10.1038/s41523-017-0053-3 29354686 PMC5765007

[B174] Yue Y. Ren L. Zhang C. Miao K. Tan K. Yang Q. (2022). Mitochondrial genome undergoes *de novo* DNA methylation that protects mtDNA against oxidative damage during the peri-implantation window. Proc. Natl. Acad. Sci. 119, e2201168119. 10.1073/pnas.2201168119 35858425 PMC9335330

[B175] Zeng X. Lu Y. Zeng T. Liu W. Huang W. Yu T. (2024). RNA demethylase FTO participates in malignant progression of gastric cancer by regulating SP1-AURKB-ATM pathway. Commun. Biol. 7, 800. 10.1038/s42003-024-06477-y 38956367 PMC11220007

[B176] Zhang Y. Duan S. Jang A. Mao L. Liu X. Huang G. (2021). JQ1, a selective inhibitor of BRD4, suppresses retinoblastoma cell growth by inducing cell cycle arrest and apoptosis. Exp. Eye Res. 202, 108304. 10.1016/j.exer.2020.108304 33080301

[B177] Zhao J. Wu S. Wang D. Edwards H. Thibodeau J. Kim S. (2024). Panobinostat sensitizes AraC-resistant AML cells to the combination of azacitidine and venetoclax. Biochem. Pharmacol. 228, 116065. 10.1016/j.bcp.2024.116065 38373594 PMC12758454

[B178] Zheng B. Fu J. Wang Y. Wu J. Wang J. Li H. (2025a). Preclinical and case series studies on the combination of venetoclax with epigenetic drugs in T-Cell acute lymphoblastic leukemia. Cancer Manag. Res. 17, 2513–2521. 10.2147/cmar.S523414 41185639 PMC12579832

[B179] Zheng Z. Lin F. Zhao B. Chen G. Wei C. Chen X. (2025b). ALKBH5 suppresses gastric cancer tumorigenesis and metastasis by inhibiting the translation of uncapped WRAP53 RNA isoforms in an m6A-dependent manner. Mol. Cancer. 24, 19. 10.1186/s12943-024-02223-4 39815301 PMC11734446

[B180] Zhou S. Zhang S. Wang L. Huang S. Yuan Y. Yang J. (2020). BET protein inhibitor JQ1 downregulates chromatin accessibility and suppresses metastasis of gastric cancer via inactivating RUNX2/NID1 signaling. Oncogenesis. 9, 33. 10.1038/s41389-020-0218-z 32157097 PMC7064486

[B181] Zhu A. X. Macarulla T. Javle M. M. Kelley R. K. Lubner S. J. Adeva J. (2021). Final overall survival efficacy results of ivosidenib for patients with advanced cholangiocarcinoma with IDH1 mutation: the phase 3 randomized clinical ClarIDHy trial. JAMA Oncol. 7, 1669–1677. 10.1001/jamaoncol.2021.3836 34554208 PMC8461552

